# Effect of faba bean-garlic intercropping on low-molecular-weight organic acids, yield components, and profitability under different spatial arrangements

**DOI:** 10.1038/s41598-026-49974-2

**Published:** 2026-04-30

**Authors:** Asmaa Hamoda, Amira A. El-Mehy, Mokhtar Dabbour, Zeinab E. Arab

**Affiliations:** 1 Department of Agronomy, Faculty of Agriculture, Benha University, P.O. Box 13736, Moshtohor, Qaluobia Egypt; 2 Crop Intensification Research Department, Field Crops Research Institute, Agricultural Research Center, Giza, Egypt; 3 Department of Agricultural and Biosystems Engineering, Faculty of Agriculture, Benha University, P.O. Box 13736, Moshtohor, Qaluobia Egypt; 4 Department of Horticulture, Faculty of Agriculture, Benha University, P.O. Box 13736, Moshtohor, Qaluobia Egypt

**Keywords:** Crop yield, Organic acid content, Intercropping systems, Land equivalent ratio, Profitability, Relative crowding coefficient, Ecology, Ecology, Environmental sciences, Plant sciences

## Abstract

A two-year field experiment was conducted to quantify the influence of spatial configuration on soil organic acids, agronomic traits, competitive indices, and economic returns in faba bean-garlic intercropping. Intercropping patterns, particularly the faba bean-garlic side-by-side arrangement (1SF:1SG), remarkably increased rhizosphere levels of ascorbic, salicylic, citric, oxalic, and total organic acids. Notably, the alternating ridge arrangement (2RF:2RG) maximized the stem and whole-plant dry weight of faba bean, whereas the 1SF:1SG pattern produced the heaviest fresh and dry bulb weights of garlic in both seasons. The highest yields were obtained from sole cropping, with 4275.20 kg ha^−1^ for faba bean and 16.07 t ha^−1^ for garlic, while the 2RF:2RG pattern was the top-performing intercrop, yielding 4004.06 kg ha^−1^ and 10.98 t ha^−1^, respectively. This pattern also optimized the land equivalent ratio and relative crowding coefficient. Garlic was the dominant competitor, exhibiting significantly greater competition ratio and aggressivity than faba bean (*p* < 0.05). Economically, the 2RF:2RG was the most profitable, achieving the maximum net returns of $3260.3 and $3980.7 ha^−1^ in the first and second seasons, respectively. Multivariate analyses, including Pearson’s correlation, PCA, radar plot, hierarchical clustering, confirmed that the 2RF:2RG produced a distinctly favorable profile for yield and competitive traits, thereby enhancing overall profitability.

## Introduction

The dual challenges of rapid global population growth and limited agricultural resources necessitate a fundamental transformation of food production systems. Achieving sustainable food security for an estimated 9–10 billion people by 2050 demands radical innovative approaches, particularly under climate change and declining land and water availability for agriculture^1^. Compounding these macro-level pressures, such as rising production costs, land prices often outpace agricultural returns per hectare, especially in regions with high amenity value. This divergence, rather than increasing production costs alone, creates an implicit opportunity cost barrier to land expansion. Consequently, landholders are compelled to enhance profitability on existing acreage instead of acquiring more land. This imperative calls for sustainable technologies that maximize resource use and improve yields from existing farmland^2^. In this context, intercropping, the practice that increases plant diversity within agricultural systems, has emerged as a key strategy for sustainable development^3–5^. Beyond diversifying food crop supply in developing countries, intercropping provides multiple ecosystem services, notably by enhancing land-use efficiency, food security, and income for smallholders^6^. However, its widespread adoption faces significant challenges. Existing agricultural value chains, policies, and knowledge systems remain predominantly designed for monocultures, creating a critical need for targeted scientific and technical support for farmers and advisors. Moreover, regional variations in pedo-climatic conditions, agricultural practices, and consumer habits require locally tailored solutions^7^. Despite these challenges, intercropping creates a more sustainable and productive system by optimizing the use of resources (nutrients, water, and light) and reducing biotic stress from pests, diseases, and weeds^8,9^, thereby achieving higher total yields than sole cropping. Among intercropping systems, legume-nonlegume combinations are the most common, driven by interspecific facilitation and complementarity^10^. A typical example is faba bean-garlic intercropping (FBGI), which provides several key agronomic benefits, including improved crop yield^11^, enhanced land productivity and resource use efficiency^12^, and increased field-level biodiversity^13^.

A key belowground mechanism facilitating legume-nonlegume intercropping is the root exudation of low-molecular-weight organic acids (e.g., ascorbic, salicylic, citric, oxalic) into the rhizosphere. These acids mobilize phosphorus from insoluble pools, chelate micronutrients (e.g., iron, zinc), alleviate aluminum toxicity under acidic conditions, and modulate rhizosphere pH and microbial communities, thereby enhancing nutrient acquisition by companion crops^14,15^. For example, organic acids from faba bean roots solubilize phosphorus utilized by intercropped garlic, reducing the demand for synthetic phosphate fertilizer^16^. Certain acids also exhibit allelopathic effects against soil-borne pathogens and weeds, lowering biotic stress in well-designed intercropping systems^13,17^. However, the influence of spatial arrangement on soil organic acid level and composition in faba bean-garlic intercropping remains poorly understood, a knowledge gap that the present study seeks to address.

The productivity of intercropping systems depends on balancing the interspecific competition and facilitation that coexist between crop species. Strategic coordination of these interactions enables the efficient use of light, water, heat, and nutrients^18,19^. This coordination is particularly substantial during the co-growth phase, when competition for limited resources intensifies within a shared space due to ecological niche differentiation^20^. Consequently, establishing a balanced and stable system is crucial for maximizing overall crop yield. A clear example of this challenge is the asymmetric competition for light in FBGI systems, which arises from their spatial structure. Taller faba bean plants capitalize on their elevated niche to capture more sunlight, boosting their biomass and grain yield^21^, whereas the subsequent shading suppresses light interception and utilization by the shorter garlic plants^22^. Therefore, optimizing the planting strategies is essential to mitigate this inherent imbalance and enhance overall intercropping yield.

In FBGI intercropping, different planting patterns directly influence interspecific relationships and resource competition. Among the various management factors, spatial arrangement, particularly row configuration, is a recognized key factor for managing this competition and enhancing yield^23^. However, the specific connection between the nutrient aggressivity of the component crops and the overall land productivity of FBGI systems is still unexplored, warranting further investigation A primary agronomic mechanism behind the yield advantage of optimal arrangement is improved radiation interception, achieved through optimized ground cove^24^. Accordingly, the chosen pattern dictates the growing space and resource access for each species, thereby directly impacting the growth and performance of the intercropped garlic^11^. The efficacy of specific patterns can be quantified using the land equivalent ratio (LER). For instance, Mohammadi et al.^11^ reported an LER greater than one, indicating higher land productivity, especially with a faba-bean-to-garlic ratio of 3:5. Similarly, in a faba bean-onion system, Abo El-Kassem and El-Shaieny^25^ identified the F3:O3 row arrangement as optimal, as it produced the highest LER, LEC, and RCC compared to the F1:O1 pattern.

Despite established traditional practices and existing evidence on the benefits of intercropping, the optimal spatial configurations for FBGI systems remain empirically undefined under Egyptian agro-ecological conditions. Consequently, landholders lack evidence-based guidance to maximize overall productivity. A critical knowledge gap also persists regarding how different row arrangements influence soil organic acids, crop growth, and overall productivity. Furthermore, a comprehensive cost-benefit analysis of FBGI systems has yet to be conducted. Therefore, this study aimed to assess the effect of spatial arrangement in an FBGI system on soil organic acids, growth, yield components, total yields, competitive indices, and profitability of the component crops. The relationships between intercropping arrangements and the measured traits (i.e., growth, yield, and competitive indices of faba bean and garlic) were also analyzed using Pearson’s correlation, principal component analysis (PCA), radar plots, and heatmap. The results of this study are expected to provide a practical framework for optimizing row arrangements, enabling farmers in similar agro-ecologies to enhance the land-use efficiency, sustainability, and profitability of legume-allium intercropping systems.

## Materials and methods

### Experimental site and meteorological conditions

**Fig. 1:**
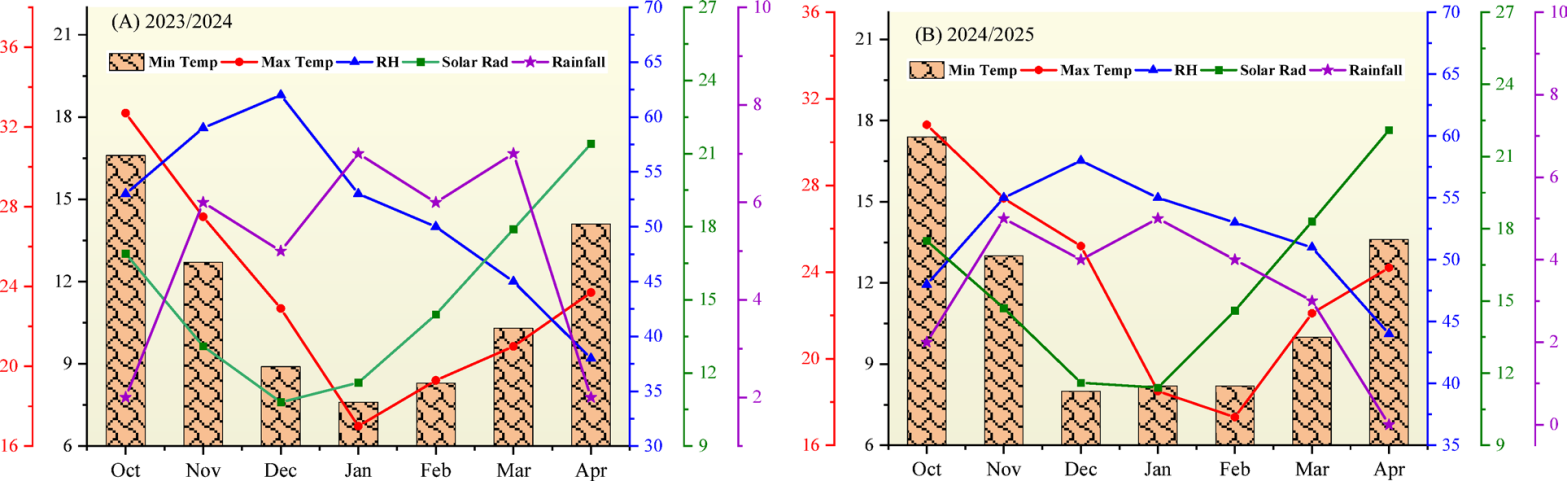
Monthly climatic data for the experimental site during the 2023/2024 and 2024/2025 growing seasons: minimum and maximum temperature (°C), relative humidity (%), solar radiation (MJ m^− 2^ day^−1^), and rainfall (mm). Data were obtained from the Central Laboratory for Agricultural Climate, Agricultural Research Center, Ministry of Agriculture and Land Reclamation, Egypt.

A 2-year field experiment was conducted during the consecutive winter seasons of 2023/2024 and 2024/2025 at the Research and Experimental Station, Faculty of Agriculture, Benha University, Egypt (31.10°E and 30.45°N). Before experiment, the soil was sampled from a depth of 0–30 cm at several randomly selected locations across the experimental site using a soil auger. These samples were composited, dried, milled, and sieved through a 2-mm mesh. Three representative subsamples were then taken for the analysis. The physical and chemical characteristics were analyzed using standard methods as previously outlined^26,27^, and the results are presented in Table 1. The analysis revealed that the soil was classified as clay-textured, with a mean alkaline pH of 8.35 and an organic matter (OM) content of 2.32%. Particle size distribution was predominantly clay (54.90%), with lower proportions of silt (24.35%) and sand (20.75%). Soil analysis indicated low salinity, with an average electrical conductivity (EC) of 0.71 dS m^−1^. The mean concentrations of available soil nitrogen (N), phosphorus (P), and potassium (K) were 28.85, 10.50, and 102.50 mg kg^−1^, respectively. The ionic composition of the soil solution was characterized by calcium (Ca^2+^: 0.61 mmol_c_ L^−1^) as the dominant cation, with lower concentrations of magnesium (Mg^2+^: 0.41 mmol_c_ L^−1^) and potassium (K^+^: 0.11 mmol_c_ L^−1^). Sulfate (SO_4_^2−^: 4.86 mmol_c_ L^−1^) was the primary anion, followed by chloride (Cl^−^: 1.53 mmol_c_ L^−1^) and nitrate (HCO_3_^−^: 1.45 mmol_c_ L^−1^). Furthermore, Fig. 1 presents the average meteorological conditions during the faba bean-garlic growth seasons (October to April) for 2023/2024 and 2024/2025. The mean maximum temperature of 23.54 °C and solar radiation of 15.45 MJ m^− 2^ day^−1^ provided sufficient photosynthetic potential, supporting the vegetative development and reproductive processes of both crops. Conversely, the lower mean minimum temperature (11.21 °C) likely influenced early growth stages and phenology. The substantially low rainfall (4.15 mm) recorded over the 7-month growing period, along with a relative humidity of 51.72%, confirm that the cropping system requires precise irrigation management to meet crop water requirements.

**Table 1:** The physical and chemical characteristics of the investigational site.

Particle size distribution (%)									
Season	Sand (%)		Silt (%)			Clay (%)		Soil texture	
2023/2024	21.0		24.0			55.0		Clay	
2024/2025	20.5		24.7			54.8		Clay	

Chemical properties						
	pH	EC (dS m^−1^)	OM (%)	Available N (mg kg^−1^)	Available P (mg kg^−1^)	Available K (mg kg^−1^)
2023/2024	8.28	0.67	2.41	28.3	10.0	100.0
2024/2025	8.41	0.74	2.23	29.4	11.0	105.0

	Soluble cations (mmol_c_ L^−1^)				Soluble anions (mmol_c_ L^−1^)				
	Ca^++^	Mg^++^		K^+^	Cl^−^		HCO_3_^–^		SO_4_^2−^
2023/2024	0.55	0.41		0.10	1.41		1.30		4.92
2024/2025	0.66	0.40		0.12	1.65		1.60		4.79

### Experimental design and setup

The experiment was laid out in a randomized complete block design with three replications. The experimental unit was a 21.6 m^2^ plot containing twelve ridges, each 3 m in length and 0.6 m in width. Data were collected exclusively from the central ridges. The treatments comprised five different spatial arrangements for intercropping faba bean and garlic, as detailed below:

1. Sole faba bean (SF): Faba bean planted on both sides of the ridge, with one plant per hill at a spacing of 25 cm apart.
2. Sole garlic (SG): Three rows of garlic planted on the ridge, with plants spaced 10 cm between hills.
3. Faba bean-garlic intercrop (2SF:1LG): Two rows of faba bean on the sides of the ridge with a central row of garlic.
4. Faba bean-garlic intercrop (1SF:1SG): One side of the ridge planted with faba bean and the other side with garlic.
5. Faba bean-garlic intercrop (2RF:2RG): A strip planting configuration with two ridges of faba bean alternating with two ridges of garlic.

**Fig. 2:**
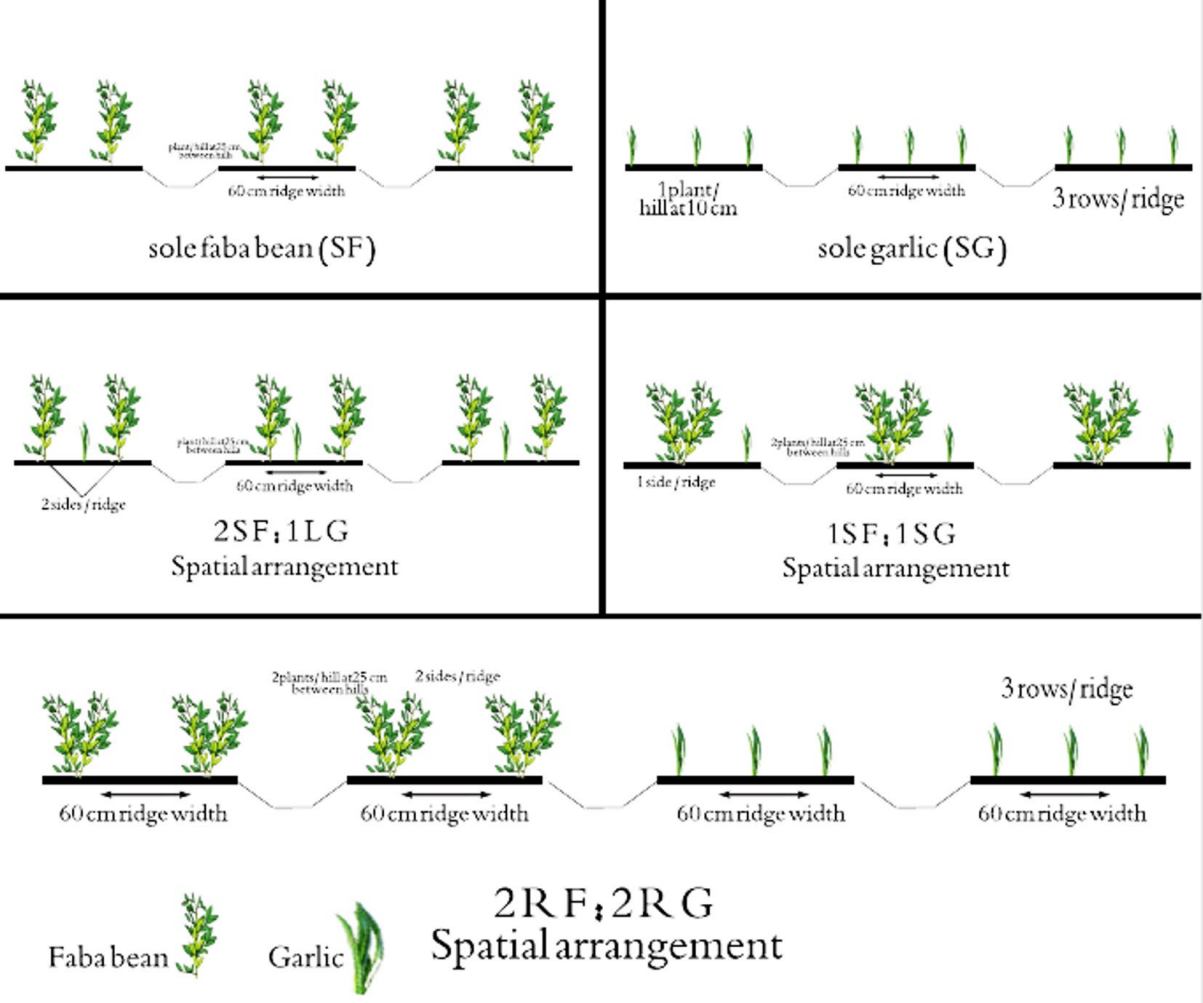
Experimental layout showing the spatial arrangements of faba bean and garlic in the intercropping system.

For all treatments, seeds were planted on 60 cm-wide ridges, with faba bean and garlic spaced at 25 cm and 10 cm between hills, respectively (Fig. 2 and Table 2). The sole crops (SF and SG) were established as monoculture controls for the assessment of intercropping performance via competitive indices and economic analysis.

**Table 2:** Plant configuration and population density of faba bean and garlic under different spatial intercropping arrangements.

Spatial arrangement	Plant spacing (cm)		Number of rows per ridge		Faba bean plants per hill	Plant density (plant.ha^−1^)	
	Faba bean	Garlic	Faba bean	Garlic		Faba bean	Garlic
SF	25	–	2	–	1 plant	133,333	–
SG	–	10	–	3	–	–	500,000
2 SF:1LG	25	10	2	1	1 plant	133,333	166,667
1SF:1SG	25	10	1	1	2 plants	133,333	166,667
2RF:2RG	25	10	2	3	2 plants	133,333	250,000

### Field practices

The field was prepared by ploughing, leveling, and forming ridges to establish a uniform seedbed before being divided into experimental plots. Calcium superphosphate (15.5% P_2_O_5_) was uniformly broadcasted across all plots at a recommended rate of 357 kg ha^−1^ before sowing. Faba bean (cv. Giza 716) seeds and garlic (cv. Balady) were purchased from the Research Institute of the Agricultural Research Center, Ministry of Agriculture and Land Reclamation, Egypt. Faba bean was sown on November 10 and 13 during the 2023/2024 and 2024/2025 seasons, respectively. To ensure bulb initiation, garlic was sown 30 days prior to faba bean in both seasons. Faba bean was harvested on April 24 and 29 in 2024 and 2025, respectively, while garlic was harvested on April 18 and 23 in the same seasons. Sesame was the preceding crop in the two seasons. Prior to sowing, faba bean seeds were inoculated with *Rhizobium leguminosarum* using gum Arabic solution (16%) as an adhesive agent. Inoculation was performed for both sole and intercropping systems. Potassium sulphate (48.8% K_2_O) was applied at a rate of 119 kg ha^−1^. Nitrogen was added 2 weeks after sowing in the form of urea (46% N) at a dosage of 50 kg N ha^−1^. Garlic was fertilized with ammonium nitrate (33.5% N) at a total recommended rate of 1071 kg ha^−1^, split equally into three applications at 30, 60, and 90 days after sowing. For intercropping treatments, this rate was proportionally adjusted based on garlic plant density. A furrow irrigation system was used in both seasons, and all other field management followed standard local recommendations.

### Data collection and measurements

#### Analysis of soil organic acids

Soil organic acids, including oxalic, citric, ascorbic, maleic, formic, and salicylic acids, were quantified following the protocol of Mimmo et al.^28^ with slight modifications. Rhizosphere soil samples were collected from the faba bean plants at 120 days after planting in the second season, then air-dried, crushed, and sieved using a 2 mm mesh. Subsequently, 5 g of soil samples was mixed with 25 mL of 0.1 mol L^−1^ phosphoric acid and centrifuged to collect the supernatant. The supernatant was filtered through a 0.45 μm cellulose membrane filter. Prior to chromatographic injection, the filtrate was acidified with ortho-phosphoric acid to a pH of ~ 2.5 to protonate and stabilize the organic acids. Analysis was performed using an Agilent 1260 series HPLC system (Agilent Technologies, Santa Clara, CA, USA) equipped with a UV-Vis detector. Separation was achieved using a reverse-phase C18 column (250 mm × 4.6 mm i.d., 5.0 μm particle size). The isocratic mobile phase consisted of 20 mM potassium dihydrogen phosphate buffer (pH 2.5). The flow rate was maintained at 1.0 mL min^−1^, the column temperature was 30 °C, and the detection wavelength was 210 nm. Quantification of specific acids was performed based on calibration curves generated from analytical standards of each target acid.

#### Growth traits of faba bean

Growth characteristics of faba bean were evaluated at 60 days after planting on randomly selected plants from each plot. Plant height (cm) was measured from the soil surface to the apex using a measuring tape. The number of branches per plant was also recorded. Chlorophyll content was estimated non-destructively using a SPAD chlorophyll meter (502-SPAD, Minolta, Japan) on the fourth leaf, following the method of Süß et al.^29^. For dry weight determination, plant samples were separated into leaves and stems, oven-dried at 70 °C for 72 h, and then weighed.

#### Yield and yield components of faba bean

At harvest, ten guarded plants were randomly sampled from the central ridges of each plot across all replications. Plant height was measured from the soil surface to the apex. The number of pods per plant was determined by counting all physiologically mature pods from the sampled plants and calculating the average. The number of seeds per pod was quantified by shelling the pods from the sampled plants, counting the total seeds, and calculating the average per pod. For the 100-seed weight (g), five random samples of 100-seed from each plot were weighed, and the mean was estimated. Seed yield was determined by weighing the total grain from each plot and converting the weight to kg ha^−1^. Biological yield representing the total above-ground dry matter, was measured from a designated subplot area and reported in kg ha^−1^.

#### Growth and yield traits of garlic

Ten plants were randomly selected from each experimental plot at 135 days after planting to assess the following growth traits: plant height (cm), number of leaves per plant, total chlorophyll content (SPAD value), fresh and dry weights of the bulbs. At harvest, yield traits of garlic were measured on randomly selected plants from each plot. Bulb diameter, number of cloves per plant and average clove weight (g) were determined. The total garlic yield was quantified from each plot and expressed in tons per hectare (t ha^−1^).

### Competitive indices and yield advantages

#### Land equivalent ratio

The land equivalent ratio (LER) was used to evaluate the productivity of the intercropping system relative to the sole crops, according to the method of Mead and Willey^30^. The LER was computed as follows:

$$LER = \frac{{Y_{{ab}} }}{{Y_{{aa}} }} + \frac{{Y_{{ba}} }}{{Y_{{bb}} }}$$

where Y_ab_ and Y_ba_ are the yields of intercropped faba bean and garlic per unit area of the intercropping system, respectively and Y_aa_ and Y_bb_ are the yields of faba bean and garlic per unit area grown as sole crops, respectively. An LER value greater than 1 indicates a yield advantage for the intercropping system.

#### Land equivalent coefficient

The land equivalent coefficient (LEC) was determined as a measure of the strength of association between the intercropped species, following the protocol of Adetiloye et al.^31^. An LEC value greater than 0.25 signifies a yield advantage.

#### Competitive ratio

The competitive ratio (CR) was calculated to quantify the competitive intensity of each species within the intercropping system, as outlined by Willey and Rao^32^. The computation of CR for each species was done as follows:

$$CR={CR}_{a}+{CR}_{b}$$

$${CR}_{a}=\frac{{LER}_{a}}{{LER}_{b}}\times\:\frac{{Z}_{ba}}{{Z}_{ab}}$$

$${CR}_{b}=\frac{{LER}_{b}}{{LER}_{a}}\times\:\frac{{Z}_{ab}}{{Z}_{ba}}$$

where CR_a_ and CR_b_ represent the competitive ratios of faba bean and garlic, respectively; Z_ab_ represents the proportion of the intercropped area allocated to faba bean; and Z_ba_ represents the proportion of the intercropped area initially allocated to garlic.

#### Aggressivity

Aggressivity (A) of faba bean and garlic was determined to quantify the competitive ability and dominance of one crop over the other in the intercropping system, as defined by Mc-Gilchrist^33^. The aggressivity of faba bean and garlic was calculated as follows:

$${Ag}_{Faba}=\frac{{Y}_{ab}}{{Y}_{aa}\:\times\:\:{Z}_{ab}}-\frac{{Y}_{ba}}{{Y}_{bb}\:\times\:\:{Z}_{ba}}$$

$${Ag}_{Garlic}=\frac{{Y}_{ba}}{{Y}_{bb}\:\times\:\:{Z}_{ba}}-\frac{{Y}_{ab}}{{Y}_{aa}\:\times\:\:{Z}_{ab}}$$

where Ag_Faba_ is the aggressivity of faba bean relative to garlic and Ag_Garlic_ is the aggressivity of garlic relative to faba bean. An A_ab_ value > 0 indicates that faba bean is the dominant competitor, while an A_ab_ value < 0 implies that garlic is dominant. A value of zero suggests that the two crops are equally competitive.

#### Relative crowding coefficient

The relative crowding coefficient (RCC) was determined to assess the relative efficiency of the intercropped faba bean and garlic in utilizing environmental resources relative to their sole crops. The RCC was computed according to the method of Dewit^34^, as follows:

$$RCC={K}_{ab}\times\:{K}_{ba}$$

$${K}_{ab}=\frac{{Y}_{ab}\:\times\:\:{Z}_{ba}}{\left({Y}_{aa}\:-\:{Y}_{ab}\right)\:\times\:\:{Z}_{ab}}$$

$${K}_{ba}=\frac{{Y}_{ba}\:\times\:\:{Z}_{ab}}{\left({Y}_{bb}\:-\:{Y}_{ba}\right)\:\times\:\:{Z}_{ba}}$$

where K_ab_ refers to the relative crowding coefficient of faba bean in association with garlic, and K_ba_ refers to the relative crowding coefficient of garlic in association with faba bean. An RCC value = 1 indicates no overall yield advantage or disadvantage. An RCC value > 1 suggests a net yield advantage and that the intercropping system is more efficient than the sole crops, whereas an RCC value < 1 implies a yield disadvantage.

#### Actual yield loss

The actual yield loss (AYL) was calculated to provide a realistic assessment of the yield loss or gain for faba bean and garlic within the intercropping system, after accounting for the actual land area they occupied. The calculation of AYL was performed according to the method of Banik^35^, as follows:

$$AYL={AYL}_{F}+{AYL}_{G}$$

$${AYL}_{F}=\left(\frac{{Y}_{ab}\times\:{Z}_{aa}}{{Y}_{aa}\times\:{Z}_{ab}}\right)-1$$

$${AYL}_{G}=\left(\frac{{Y}_{ba}\times\:{Z}_{bb}}{{Y}_{bb}\times\:{Z}_{ba}}\right)-1$$

where AYL_F_ and AYL_G_ represent the actual yield loss of faba bean and garlic, respectively; and Z_aa_ and Z_bb_ represent the sown proportion (i.e., the fraction of land area) in the sole crops, which is conventionally 1 (or 100%). For an individual crop, a positive AYL value (AYL_F_ or AYL_G_) signifies a yield gain in intercropping compared to its sole crop, while a negative value indicates a yield loss. A positive overall AYL signifies that the yield benefits for the system collectively outweigh the losses.

#### Economic analysis

An economic analysis was conducted to assess the financial viability of the intercropping systems compared to sole cropping. The total production cost for each treatment was calculated, encompassing variable inputs (seeds, fertilizers, pesticides) and fixed costs (land rent, machinery use, and labor). The marketable yield for faba bean and garlic was valued using the prevailing local market prices from the respective harvest seasons. The economic indices were calculated as follows:

$$Gross\:return=\left(Faba\:bean\:yield\times\:price\right)+\:\left(Garlic\:yield\times\:price\right)$$

$$Net\:return=Gross\:return-\:Total\:cost\:of\:cultivation$$

$$BCR=\frac{Gross\:return}{Total\:cost\:of\:cultivation}$$

where BCR is the benefit-cost ratio which measures the economic efficiency of a system by comparing the returns per unit of cost incurred. A BCR greater than 1.0 indicates that the system is economically feasible and profitable, as the returns exceed the costs. Conversely, a BCR less than 1.0 signifies a net financial loss. Among treatments, the intercropping system achieving the highest BCR and net return is considered the most economically advantageous and is recommended for adoption.

#### Statistical analysis

The results are presented as means ± standard deviation of three experimental replications. A one-way ANOVA was performed using the COSTAT-V6.311 software package (Cohort software, Berkeley, CA, USA) to assess the significance of differences among treatment means. Tukey’s post-hoc test was applied at *p* < 0.05. Furthermore, a Pearson’s correlation, principal component analysis (PCA) radar plot, and heatmap were performed using OriginPro 2023b software (OriginLab Corporation, MA, USA) to visualize associations among the studied traits and treatments.

## Results and discussion

### Total organic acid in the soil

**Fig. 3:**
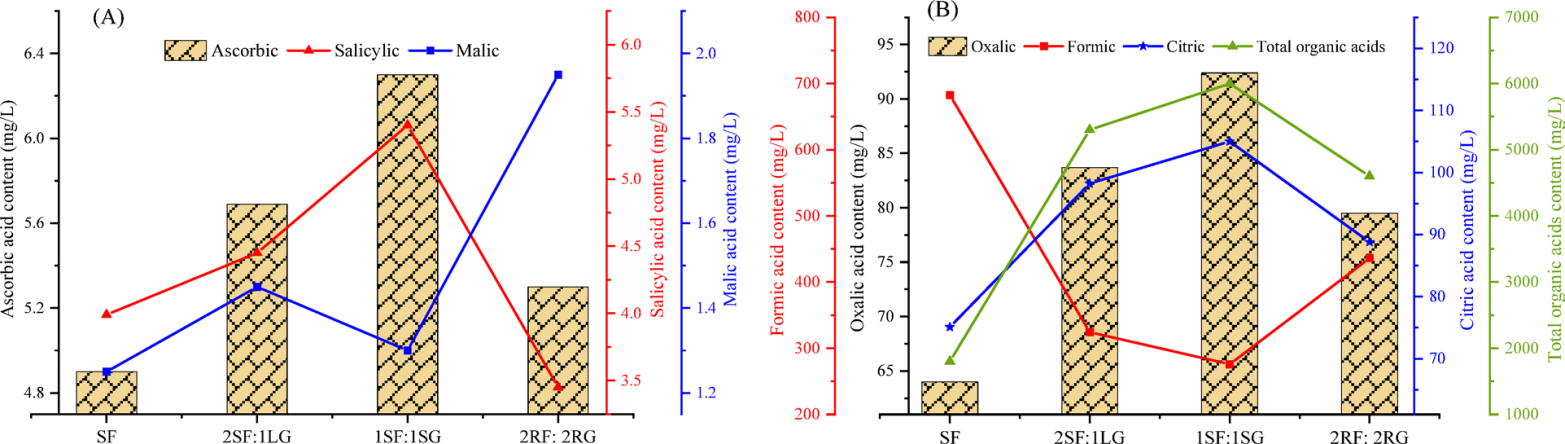
Effect of spatial arrangement on soil organic acid contents in faba bean-garlic intercropping systems.

Total organic acids are crucial for soil fertility and nutrient bioavailability. They facilitate the solubilization and release of nutrients from minerals, making them available for plant uptake^36^. The results indicated that the spatial arrangement of faba bean and garlic notably influenced the soil organic acid (OA) contents (Fig. 3). The highest concentrations of ascorbic and salicylic acids (6.30 and 5.40 mg L^−1^, respectively) were detected in the 1SF:1SG arrangement, followed by 2SF:1LG (5.69 and 4.45 mg L^−1^, respectively) (Fig. 3A). In the faba bean rhizosphere, the 2RF:2RG treatment yielded the highest values of malic acid (1.95 mg L^−1^). However, the lowest concentrations of ascorbic and malic acids were observed in sole-cropped faba bean, whereas the 2RF:2RG pattern produced the minimal salicylic acid content. These results are in line with the findings of Zhang et al.^37^, who found that malic acid (185.71%), oxalic acid (442.86%), and lactic acid (31.61%) were significantly higher levels in intercropping systems than sole-cropped soybean, although citric acid was not detected in soybean root exudates. Sole faba bean had the highest soil formic acid concentration in the rhizosphere, followed by the 2RF:2RG treatment (Fig. 3B). This indicated that faba bean, whether grown as a sole crop or in alternate rows with garlic, released substantial amounts of formic acid into the rhizosphere. The observed result may be due to the fact that formic acid concentration was strongly influenced by the composition of root exudates. Factors such as plant genotype and developmental stage can substantially impact the rate of OA production and release in the rhizosphere^38^. Regarding oxalic and citric acid, their highest concentrations in the faba bean rhizosphere were observed in the 1SF:1SG (92.4 and 105 mg L^−1^, respectively). These values were close to those in the 2SF:1LG (83.7 and 98.23 mg L^−1^) and 2RF:2RG (79.5 and 88.8 mg L^−1^) treatments. In contrast, the lowest concentrations of both acids were found in the sole faba bean. Comparable findings have also been reported by Ritota et al.^39^ who found formic, citric and malic acids in the analyses of several red and white garlic cultivars. Furthermore, Zhang et al.^40^ observed that intercropping three aromatic plants in a pear orchard increased OA content relative to the control.

Moreover, intercropping consistently resulted in higher total OA content than sole cropping. The highest OA content in the faba bean rhizosphere (6000 mg L^−1^) was recorded in the 1SF:1SG intercrop system (Fig. 3B). This was followed by the 2SF:1LG arrangement (5300 mg L^−1^) and the 2RF:2RG alternating arrangement (4600 mg L^−1^). Nonetheless, sole-cropped faba bean yielded the lowest value (1800 mg L^−1^). These findings suggest that the 1SF:1SG system fostered the most intensive rhizosphere interactions. The elevated organic acid levels are likely linked to the transformation of the faba bean rhizosphere into a more biochemically active environment in intercropping systems, particularly in the 1SF:1SG arrangement. This transformation, driven by intensified plant-plant and plant-microbe interactions, may have promoted the significant accumulation of these acids. A key mechanism is the complementary root exudation profiles of faba bean and garlic. The combination of their distinct organic acids forms a diverse mixture of exudates, creating a synergistic effect that enhances nutrient mobilization and stimulates a broader consortium of organic acid-producing microbes than either sole crop^41^. Abdel-Wahab and Abdel-Wahab^17^ also found that intercropping systems substantially increased soil organic acid content in the faba bean rhizosphere.

Generally, changes in these acids, including formic, oxalic, citric, ascorbic, salicylic, and malic acid, are largely influenced by root exudation patterns, soil pH, and nutrient cycling within the more diversified environment of an intercrop. Additionally, the concentration of organic acids in soil is affected by the level of biological activity, which governs their synthesis and degradation. For instance, Mimmo et al.^28^ noted variations in citric and malic acids extracted from microbially active soil and attributed these variations differences in microbial activity. Consistent with these outcomes, Zhang et al.^37^ reported that the total OA content in soybeans from intercropped systems markedly exceeded that of sole-cropped soybeans, whereas the OA content in intercropped maize was not significantly affected compared to sole maize.

### Growth traits of faba bean

Analysis of variance (ANOVA) indicated that spatial arrangement had a highly significant (*p* < 0.01) effect on most growth traits, including plant height, total chlorophyll content, stem dry weight, and whole-plant dry weight (Table 3). Notably, leaf dry weight was unaffected (*p* > 0.05) in both seasons. Furthermore, in the second season, the significant effect on plant height was lost, and branch number also showed no response to the treatments.

**Fig. 4:**
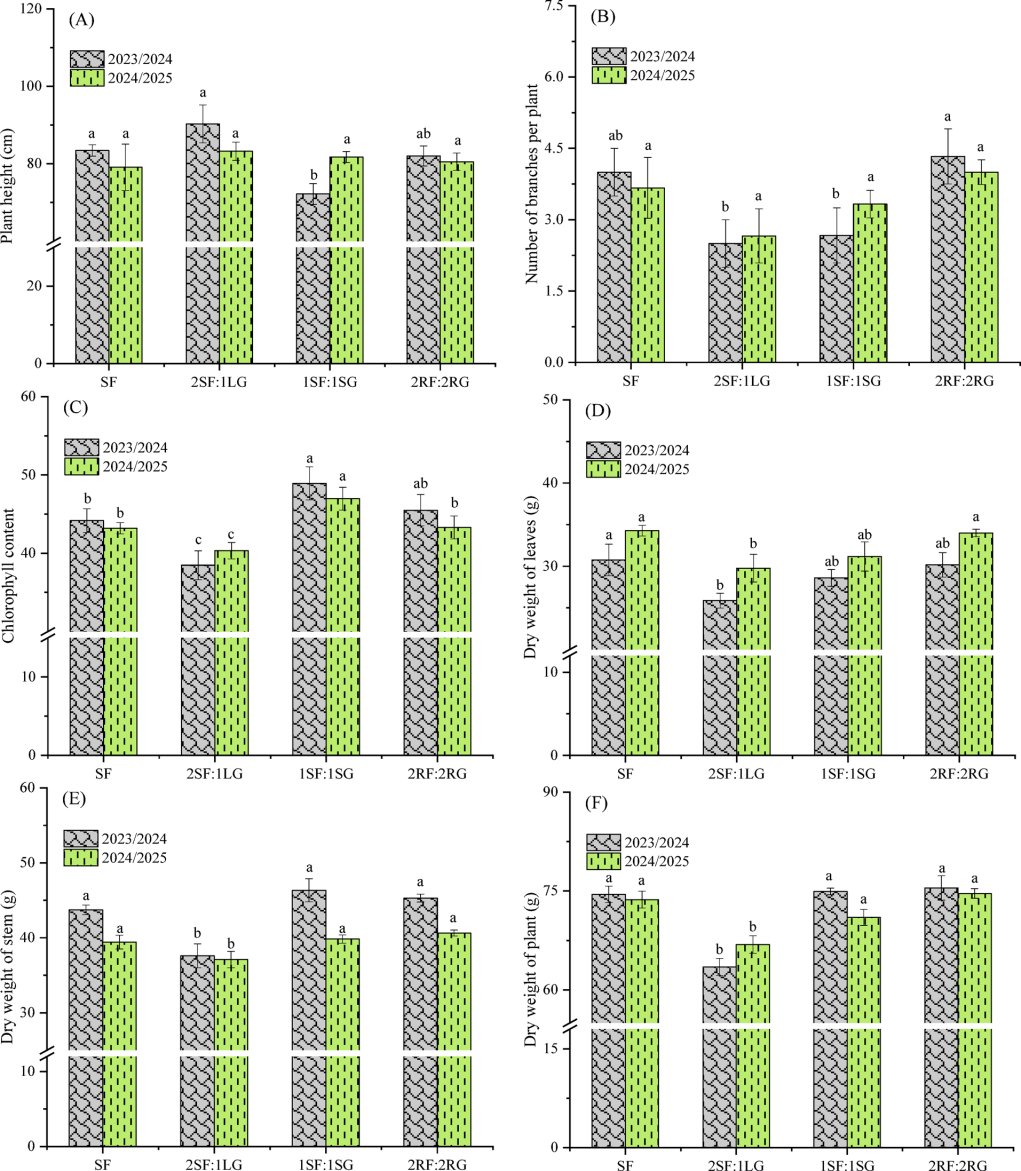
Effect of spatial arrangements on the growth traits of faba bean. Error bars represent the standard deviation of three determinations. Different letters on bars denote significant differences based on Tukey’s test at *p* < 0.05.

Figure 4A shows that the 2SF:1LG spatial arrangement produced the greatest plant height (90.27 cm) in the first season, a value that was statistically similar to those of the sole faba bean and 2RF:2RG treatments but significantly greater than that of the 1SF:1SG arrangement (*p* < 0.05). In the second season, however, plant height did not differ significantly among the spatial arrangements. Contrarily, the shortest plants were observed in the 1SF:1SG arrangement (72.20 cm) in the first season and SF (79.10 cm) in the second season. The greater plant height observed in the 2SF:1LG is linked to the spatial dynamics of root and canopy interaction. This effect is mainly due to increased competition for light; planting faba bean in both sides of the ridge likely created a more shaded environment, stimulating internode elongation and consequently increasing plant height. Increased stem elongation is a known plant mechanism for enhancing the capture efficiency of solar radiation^42^. These results are in good accordance with those obtained by Farghly et al.^43^, who reported that a 3 faba bean:1 onion intercropping system resulted in the tallest plants, with no significant difference relative to the sole faba bean. Furthermore, the highest number of branches per plant (4.33 and 4.00 in the first and second seasons, respectively) was recorded in the 2RF:2RG arrangement, followed by the sole faba bean (4.00 and 3.67), whereas the lowest number (2.55 and 2.66) was noted in the 2SF:1LG (Fig. 4B). Such findings were primarily attributable to reduced intraspecific competition for light and soil resources among faba bean plants in the 2RF:2RG pattern. This alleviated shade stress and lowered auxin-mediated apical dominance, allowing for greater cytokinin activity and resource allocation to lateral buds, thereby promoting branching. These outcomes suggest that the 2SF:1LG intercropping arrangement, where the garlic roots are in close proximity to faba bean roots, may not be optimal for branch development. This indicates that maintaining a certain spatial distance between the root systems of faba bean and garlic could be beneficial. Moreover, spatial arrangements significantly affected the SPAD readings of faba bean (Fig. 4C). Specifically, all intercropping arrangements, except for 2SF:1LG, improved the total chlorophyll content compared to the sole crop. The 1SF:1SG arrangement (faba bean intercropped with garlic) resulted in the maximum SPAD value (48.93 and 46.97, respectively in the first and second seasons), significantly surpassing all other spatial arrangements. Such enhancement was mainly ascribed to enhanced light interception, stemming from spatial niche differentiation and an optimized light distribution within the canopy. This was probably also due to enhanced nutrient availability, potentially resulting from higher total organic acid contents observed in the faba bean rhizosphere (Fig. 3). Moreover, Yang et al.^44^ indicated that intercropping faba bean with wheat significantly increased the contents of chlorophyll a, chlorophyll b, total chlorophyll (a + b), and the chlorophyll (a/b) ratio compared to a faba bean monoculture. Comparable results have also been documented in literature^12,17^.

Among the spatial arrangements, sole-planting of faba bean resulted in the highest leaf dry weight per plant (30.76 and 34.27 g in the first and second seasons, respectively) (Fig. 4D). This was followed by the arrangement of 2FR:2RG, which yielded 30.17 and 34.0 g, with no significant difference observed between these two treatments (*p* > 0.05). However, the lowest dry weight of leaf per plant (25.87 and 29.77 g) was recorded in the 2SF:1LG intercrop. This reduction may be attributed to intense aboveground competition from faba beans planted on both sides of the ridge, combined with a central row of garlic, as well as belowground competition from the closely intertwined root systems of both species in the 2FS:1LG spatial arrangement. Agili et al.^45^ also attributed biomass suppression in intercropping systems to the impacts of cumulative competition. Concerning stem dry weight per plant (Fig. 4E), the 1SF:1SG and 2RF:2RG arrangements exhibited the heaviest stems, followed by sole planting of faba bean, with no significant difference detected among these three treatments. In contrast, 2FS:1LG significantly reduced (*p* < 0.05) the stem dry weight compared to the other arrangements in both growing seasons. These results suggest that the 2SF:1LG arrangement constituted a high-density planting system that exceeded critical threshold of competition intensity, inducing severe asymmetric competition. This configuration concentrated intense intraspecific competition among faba bean rows on the same ridge for soil resources, compounded by interspecific competition from the central garlic row. The resulting high-density stress likely triggered a shade avoidance response, prioritizing stem elongation for light capture over biomass accumulation, resulting in taller plants (as shown in Fig. 4A) but with thinner, lighter stems of reduced dry matter. Figure 4F also shows that faba bean-garlic intercropping in the 2RF:2RG or 1SF:1SG arrangements resulted in plant dry weights comparable to sole faba bean in both seasons, with no significant differences observed. Specifically, the heaviest plants (75.47 and 74.63 g) were found in the 2RF:2RG row configuration in 2023/24 and 2024/25 season, respectively. Nonetheless, the 2SF:1LG configuration significantly reduced plant dry weight of faba bean relative to other intercropping patterns, recording 63.47 and 66.87 g in the first and second seasons, respectively. These differences may be explained by the distinct competitive dynamics of each spatial arrangement. The 2RF:2RG configuration may have enhanced plant dry weight through superior light interception and resource acquisition efficiency. Conversely, the 2SF:1LG arrangement may intensify both intra- and interspecific competition, thereby reducing individual plant dry weight compared to sole faba bean, despite potentially augmenting overall system yield. In line with these results, Galanopoulou et al.^46^ indicated that altering the row configuration of intercropping systems significantly influences both competitive interactions and dry matter production.

**Table 3:** Analysis of variance for the effect of spatial arrangements on growth traits of faba bean. *, **, *** = Significant at *p* ≤ 0.05, *p* ≤ 0.01, and *p* ≤ 0.001, respectively, ns = non-significant.

Source of variance	d.f	Plant height	Number of branches	Chlorophyll content	Leaf dry weight	Stem dry weight	Plant dry weight
1st season (2023/2024)							
Blocks	2	3.062^ns^	0.187^ns^	0.636^ns^	0.547^ns^	0.180^ns^	0.45^ns^
Arrangement	3	166.43**	2.57*	56.94**	14.37*	45.86**	39.54**
Error	6	12.37	0.326	4.52	2.36	2.79	2.18
F-test		13.44	7.89	12.59	6.06	24.69	18.17
*p* value		0.0045	0.0166	0.0053	0.0300	0.0009	0.0021
CV (%)		4.29	16.92	4.80	5.33	3.87	2.05
2nd season (2024/2025)							
Blocks	2	39.57^ns^	0.26^ns^	0.09^ns^	1.35^ns^	1.68*	0.28^ns^
Arrangement	3	9.263^ns^	0.97^ns^	22.16**	14.46*	6.91*	36.36**
Error	6	43.34	0.43	1.94	1.71	0.28	1.99
F-test		0.213	2.23	11.41	8.44	24.17	18.22
*p* value		0.883	0.1850	0.0068	0.0142	0.0009	0.0020
CV (%)		8.11	19.30	3.21	4.05	1.36	1.97

### Yield and yield components of faba bean

ANOVA exhibited that the influence of spatial arrangement on yield components varied by season (Table 4). Its effect was highly significant (*p* < 0.001) on seed yield and biological yield in the first season, and on 100-seed weight in the second season. In contrast, the number of seeds per pod remained unaffected by the planting pattern in both seasons.

**Fig. 5:**
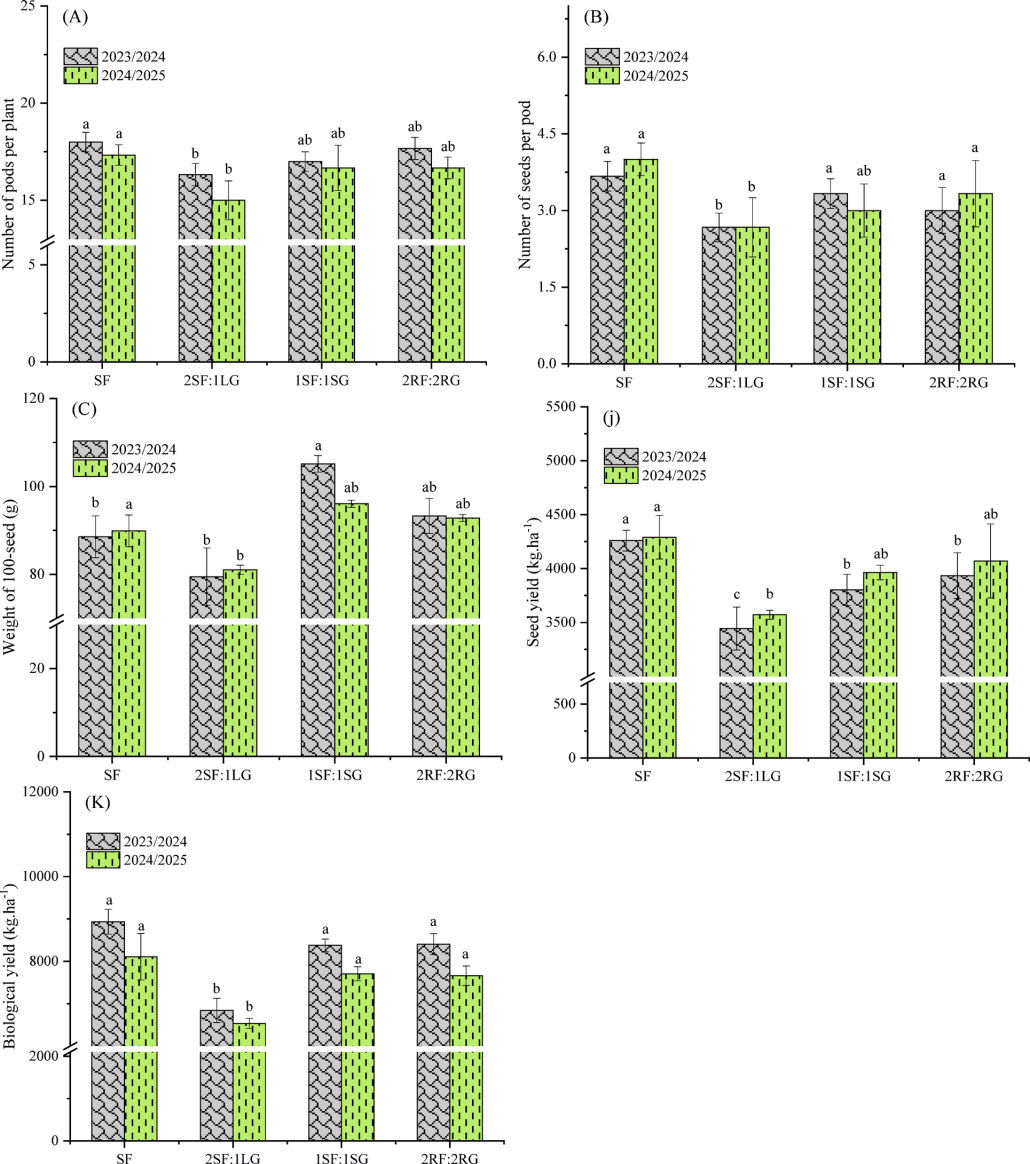
Effect of spatial arrangements on the yield and yield components of faba bean. Error bars represent the standard deviation of three determinations. Different letters on bars denote significant differences based on Tukey’s test at *p* < 0.05.

The results indicated that the 2SF:1LG intercropping arrangement substantially reduced all yield components (i.e., number of pods per plant, number of seeds per pod, and 100-seed weight) in both seasons (*p* < 0.05) (Fig. 5A–C). This reduction is primarily due to intense intra- and inter-specific competition between companion crops, which impaired chlorophyll content and dry weight accumulation (as shown in Fig. 4C–F), ultimately reducing yield components compared to other arrangements. Remarkably, the faba bean sole crop, free from interspecific competition, recorded the highest number of pods per plant (18.00 and 17.33, respectively) and number of seeds per pod (3.67 and 4.00, respectively) in the first and second seasons, performing similarly to the 2RF:2RG intercropping pattern. Furthermore, the heaviest 100-seed weight was observed in the 1SF:1SG arrangement (105.13 and 96.07 g), a result comparable to that of the 2RF:2RG pattern and significantly higher than that of the 2SF:1LG arrangement (79.46 and 81.03 g) in both growing seasons. These outcomes are consistent with the established literature on spatial arrangement in intercropping systems. For example, El-Shamy and Abd El-Aty^47^ noted that intercropping pattern of garlic and faba bean significantly affected the yield components of faba bean. Moreover, the results revealed that the maximum seed yield was produced by the sole-cropped faba bean. This represented an increase of 23.69%, 12.04%, and 8.26% in the 2023/24 season, and 27.87%, 15.24%, and 12.19% in the 2024/25 season, over the yields achieved with the 2SF:1LG, 1SF:1SG and 2RF:2RG arrangements, respectively (Fig. 5D). These results were consistent with the findings of number of pods per plant and number of seeds per pod (Fig. 5A and B). The observed increases are probably attributable to the earlier planting of garlic (30 days before faba bean), which allowed it to establish and pre-emptively capture available resources prior to the introduction of faba bean crop in the intercropping system. Additionally, the duplicate faba bean density per hill may have induced competitive stress within the root systems, which further decreased the yield of the intercropped faba bean. Zeid and Komeil^48^ also found that an opposite-side ridge intercropping method yielded 18.9% more pod weight per plant than a same-hill intercropping method. Notably, the 2RF:2RG spatial arrangement achieved the highest seed yield (4259.41 and 4290.98 kg ha^−1^ in the first and second seasons, respectively) among the intercropping treatments, closely approximating the performance of the sole faba bean in both seasons. This yield stability under an alternating ridge system suggests that the 2RF:2RG configuration effectively mitigated interspecific competition by maintaining an optimal balance between canopy light interception and root zone partitioning for both crops. Parimaladevi et al.^49^ demonstrated that a 2:2 maize-legume system maximized radiation use efficiency and minimized belowground competition, leading to sustained yield levels. Furthermore, Fig. 5E exhibits that the biological yield of faba bean did not vary significantly among the spatial arrangements across both seasons, except for the 2SF:1LG arrangement. Sole-cropped of faba bean produced the highest biological yield (8934.99 and 8107.47 kg ha^−1^ in the first and second seasons, respectively), followed by the 2RF:2RG in first season and 1SF:1SG in second season. However, the 2SF:1LG intercropping spatial arrangement yielded the lowest values (6840.83 and 6531.83 kg ha^−1^ in the first and second season, respectively). This implies that intercropping reduced biological yield relative to the sole crop, a result attributed to increased interspecific competition. Notably, a substantial positive association was observed between biological yield and the dry weight of leaves, stems, and total plant (as shown in Fig. 4D–F). These results are in accordance with previous studies, for instance, Oskoii et al.^50^ recorded the lowest biological yield in a 2:1 strip arrangement of maize and faba bean. Likewise, Bekele et al.^51^ noted that sole-planted faba bean produced the highest aboveground biomass (*p* < 0.05), followed by 2:1 faba bean-to-wheat ratio, while the lowest yield was obtained with a 1:2 faba bean-to-wheat planting ratio.

**Table 4:** Analysis of variance for the effect of spatial arrangements on yield and yield components of faba bean. *, **, *** = Significant at *p* ≤ 0.05, *p* ≤ 0.01, and *p* ≤ 0.001, respectively, ns = non-significant.

Source of variance	d.f	Pod number per plant	Seed number per pod	100-seed weight	Seed yield	Biological yield
1st season (2023/2024)						
Blocks	2	0.187^ns^	0.021^ns^	8.86^ns^	84727.4*	26577.96^ns^
Arrangement	3	1.638*	0.556^ns^	342.66**	341778.34***	2447661.8***
Error	6	0.326	0.159	25.27	10100.51	74092.95
F-test		5.021	3.478	13.560	33.83	33.03
*p* value		0.0448	0.0906	0.0044	0.0004	0.0004
CV (%)		3.31	12.62	5.48	3.60	3.34
2nd season (2024/2025)						
Blocks	2	2.08^ns^	3.00**	4.03^ns^	40566.29^ns^	31490.23^ns^
Arrangement	3	2.97^ns^	0.97^ns^	125.04***	743475.96*	1380190.2**
Error	6	0.97	0.22	3.77	88419.52	122252.02
F-test		3.06	4.37	33.15	8.41	11.28
*p* value		0.1133	0.0590	0.0004	0.0144	0.0070
CV (%)		6.00	14.50	2.16	7.26	4.65

### Growth traits of garlic

ANOVA showed that the effect of spatial arrangement on the measured traits varied significantly between seasons (Table 5). In the first season, treatments had a highly significant effect on plant height and fresh bulb weight (*p* < 0.001), a significant effect on chlorophyll content and dry bulb weight (*p* < 0.05), and no significant effect on leaf number. Conversely, during the second season, the treatments exerted a highly significant effect on chlorophyll content and dry bulb weight (*p* < 0.001), a significant effect on plant height and leaf number (*p* < 0.05), and no significant effect on fresh bulb weight.

**Fig. 6:**
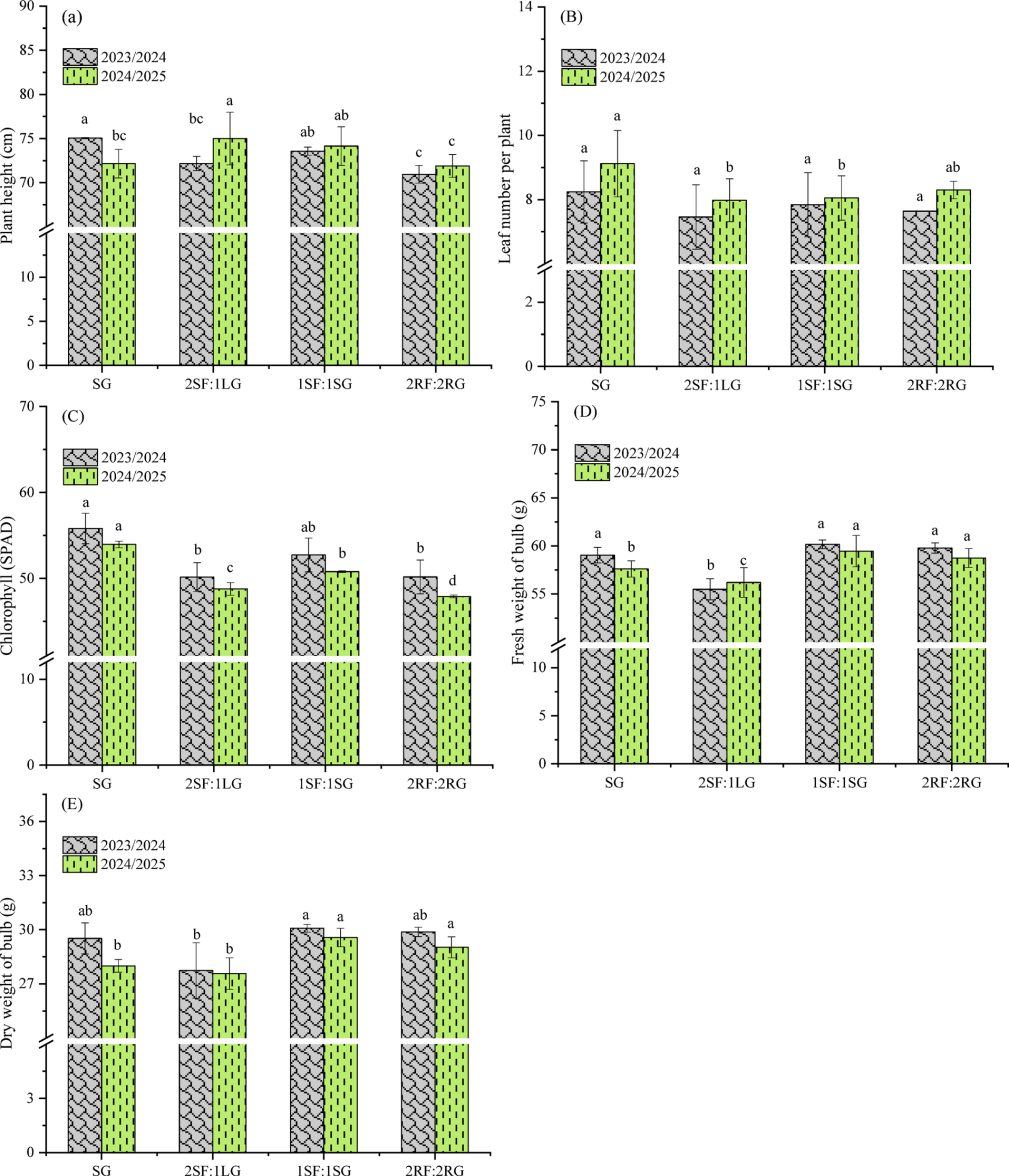
Effect of spatial arrangements on the growth traits of garlic. Error bars represent the standard deviation of three determinations. Different letters on bars denote significant differences based on Tukey’s test at *p* < 0.05.

Figure 6A demonstrates that the spatial configuration of faba bean and garlic intercrops had a significant influence on plant height across both growing seasons. Among the tested arrangements, the 2SF:1LG pattern consistently produced the tallest plants, with heights of 75.04 and 75.00 cm in the first and second seasons, respectively, followed closely by the 1SF:1SG arrangement. In contrast, the alternating ridge pattern (2RF:2RG) resulted in the most suppressed plant growth, producing the lowest mean heights of 70.95 and 71.90 cm in both seasons. This suppression may be ascribed to intensified inter- and intra-competition between plants for photosynthetic resources, a condition exacerbated by the shading canopy of taller faba bean plants. Comparable outcomes were also reported^47^. A different trend, however, was observed for leaf number per plant (Fig. 6B). Sole-cropped garlic (monoculture) produced a greater average number of leaves per plant (8.24 and 9.12 in the first and second seasons) compared to the 2SF:1LG intercrop (7.46 and 7.98, respectively). Although this represents a measurable decrease under intercropping, statistical analysis confirmed that the difference was not significant in either season. These observations are in good harmony with the results of Farghly et al.^43^, suggesting that while certain spatial arrangements promote height, they may impose a non-significant constraint on leaf development in garlic. Concerning total chlorophyll content (Fig. 6C), sole garlic exhibited a significantly higher (*p* < 0.05) chlorophyll content (55.81 and 53.95 across both successive seasons) than in most intercropping configurations. The exception was the 1SF:1SG configuration in the first season, which showed no significant difference (*p* > 0.05). The lowest values were observed in the 2SF:1LG and 2RF:2RG arrangements, with values of 50.15 and 47.90 in the first and second seasons, respectively. This pattern can be attributed to interspecific shading by the taller faba bean canopy, which reduced the availability of photosynthetically active radiation for the understory garlic. The resulting low-light conditions likely triggered a photo acclimation response, involving the downregulation of chlorophyll synthesis and a reallocation of resources to improve metabolic efficiency^24,52^. Therefore, the lower chlorophyll content in intercropped garlic acted as a direct biomarker of light competition stress. Comparable conclusions were also documented by Parimaladevi et al.^49^. For fresh and dry weight of garlic bulb (Fig. 6D and E), all spatial configurations, with the exception of the 2SF:1LG arrangement, were statistically similar in first season. Notably, the 1SF:1SG pattern emerged as optimal, delivering the highest fresh bulb weight (60.17 and 59.46 g in the first and second seasons, respectively) and dry bulb weight (30.08 and 29.57 g, respectively). Such pattern likely minimized interspecific competition for resources, providing garlic with less obstructed access to light, thereby reducing shading stress and below-ground interference from the faba bean, and improving access to soil nutrients and water. This favorable environment enhanced carbon assimilation and promoted the efficient partitioning of photoassimilates toward bulb development, which was in agreement with previous reports by Agili et al.^45^.

**Table 5:** Analysis of variance for the effect of spatial arrangements on growth of garlic. *, **, *** = Significant at *p* ≤ 0.05, *p* ≤ 0.01, and *p* ≤ 0.001, respectively, ns = non-significant.

Source of variance	d.f	Plant height	Leaf number per plant	Chlorophyll content	Fresh weight of bulb	Dry weight of bulb
1st season (2023/2024)						
Blocks	2	1.046^ns^	0.19^ns^	3.57^ns^	0.79^ns^	1.36^ns^
Arrangement	3	9.373***	0.34^ns^	21.61*	13.73***	3.43*
Error	6	0.27	0.92	3.38	0.52	0.62
F-test		33.98	0.36	6.39	26.17	5.49
*p* value		0.0004	0.781	0.026	0.0008	0.037
CV (%)		0.72	12.31	3.52	1.23	2.69
2nd season (2024/2025)						
Blocks	2	16.32***	1.76**	0.49*	6.25***	1.34***
Arrangement	3	6.89**	0.82*	21.62***	6.08***	2.53***
Error	6	0.55	0.09	0.08	0.158	0.05
F-test		12.46	8.50	266.18	38.47	51.42
*P* value		0.005	0.01	0.0001	0.0003	0.0001
CV (%)		1.01	3.71	0.56	0.68	0.77

### Yield and yield components of garlic

ANOVA revealed that, across both growing seasons, the effect of spatial arrangements on yield components showed a consistent and highly significant trend for final productivity (Table 6). Garlic yield was strongly enhanced, exhibiting a highly significant response (*p* < 0.001) in each season. This increase in yield was associated with a significant increase in the number of cloves per bulb, which was significant (*p* < 0.05) in the first season and highly significant (*p* < 0.001) in the second season. In contrast, individual clove weight and overall bulb diameter remained unaffected, showing no significant response to the treatments in either season.

**Fig. 7:**
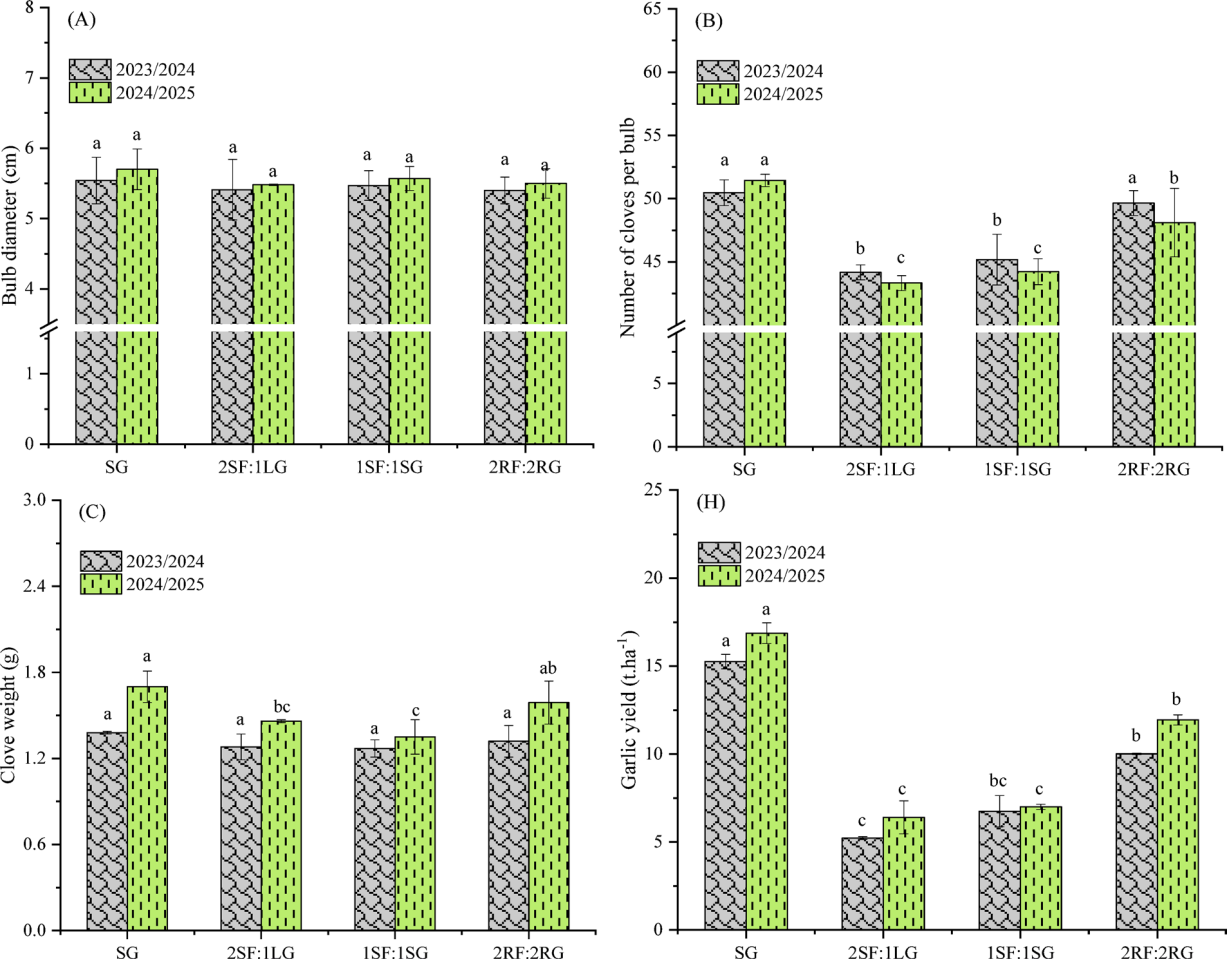
Effect of spatial arrangements on the yield and yield components of garlic. Error bars represent the standard deviation of three determinations. Different letters on bars denote significant differences based on Tukey’s test at *p* < 0.05.

The sole planting of garlic increased the bulb diameter (5.54 and 5.70 cm) compared to intercropping 2RF:2RG (5.40 cm) in the first season and 2SF:1LG (5.48 cm) in the second season (Fig. 7A). This increment, however, was not statistically significant in either season. Moreover, sole-cropped garlic yielded the highest number of cloves per bulb (40.47 and 51.44) (Fig. 7B), which were significantly greater (*p* < 0.05) than those in the 2SF:1LG and 1SF:1SG arrangements in both seasons. This implies that monoculture may have provided the optimal environment for garlic, maximizing photosynthetic assimilation (source strength) and allowing the plants to allocate a greater proportion of photoassimilates toward clove initiation and development (sink strength). The lowest clove number was consistently recorded for the 2SF:1LG system (44.18 and 43.33). The results also showed that average clove weight was not significantly influenced by spatial arrangement in the first season (Fig. 7C). In the second season, however, sole cropping produced significantly heavier (*p* < 0.05) cloves than the 2SF:1LG and 1SF:1SG arrangements. The initial non-significance suggests that compensatory physiological mechanisms or favorable early-season conditions buffered the initial impact of competition on clove filling. The significant effect observed in the second season, however, reveals that under sustained competitive pressure, the cumulative resource deficit ultimately compromised the sink-filling capacity of the bulb, reducing the dry matter allocated per clove. This pattern highlights that clove weight is affected hierarchically and may only show significant responses once competition exceeds a critical threshold of intensity or duration. Earlier studies, such as those by Farghly et al.^43^ and El-Shamy and Abd El-Aty^47^, indicated that intercropping systems had a significant effect on the average weight of garlic cloves. The spatial arrangement of garlic and faba bean significantly affected bulb yield in both seasons (Fig. 7D). As anticipated, the sole-cropped garlic yielded the highest values (15.26 and 16.87 t ha^−1^ in first and second seasons, respectively). This result highlights the fundamental role of unimpeded plant density and resource access, confirming that yield is optimized in the absence of interspecific competition. The alternating ridge arrangement (2RF:2RG), with yields of 10.01 and 11.94 t ha^−1^, achieved the second-highest productivity. This configuration promoted a more favorable canopy architecture for garlic plants by providing more space and reducing shading from the taller faba bean plants, thereby contributing to higher garlic yield. Contrarily, the 2SF:1LG arrangement produced the lowest yield (5.22 and 6.40 t.ha^−1^) in both seasons. This decrease was linked to asymmetric and intensified competition. The spatial geometry placed the single garlic row in direct and sustained competition with two dense faba bean rows, creating a highly competitive microenvironment that severely restricted access to photosynthetically active radiation, water, and key nutrients like nitrogen and phosphorus, leading to poor bulb development. Comparable observations have previously been documented^12,47^.

**Table 6:** Analysis of variance for the effect of spatial arrangements on yield and yield components of garlic. *, **, *** = Significant at *p* ≤ 0.05, *p* ≤ 0.01, and *p* ≤ 0.001, respectively, ns = non-significant.

Source of variance	d.f	Bulb diameter	Clove number per bulb	Clove weight	Garlic yield
1st season (2023/2024)					
Blocks	2	0.159^ns^	2.10^ns^	0.02*	0.07^ns^
Arrangement	3	0.011^ns^	29.80**	0.007^ns^	13.61***
Error	6	0.07	1.42	0.002	0.302
F-test		0.16	0.16	3.95	44.97
*p* value		0.916	0.916	0.07	0.0002
CV (%)		4.92	4.92	3.31	6.42
2nd season (2024/2025)					
Blocks	2	0.12*	5.76*	0.04*	0.97*
Arrangement	3	0.03^ns^	41.89***	0.07**	71.69***
Error	6	0.014	1.05	0.003	0.12
F-test		2.14	39.79	18.88	584.38
*p* value		0.196	0.0002	0.002	0.0001
CV (%)		2.12	2.19	3.98	3.31

### Competitive relationships

#### Land equivalent ratio and coefficient and competitive ratio

ANOVA results (Table 7) indicated that spatial arrangement significantly influenced most competitive indices. Specifically, RY_Garlic_, LER, LEC were highly significant (*p* < 0.001) in both seasons. RY_Faba_ also showed a significant increase at *p* < 0.001 and *p* < 0.01 in the first and second seasons, respectively. In contrast, CR_a_, CR_b_, and overall CR were not significantly affected by spatial arrangement in either season.

**Fig. 8:**
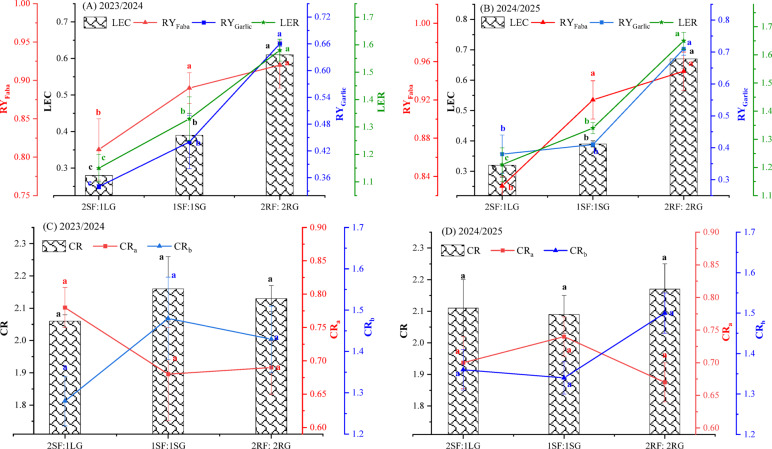
Effect of spatial arrangements on the land equivalent ratio and coefficient and competitive ratio in garlic and faba bean intercropping system. Error bars represent the standard deviation of three determinations. Different letters on bars denote significant differences based on Tukey’s test at *p* < 0.05.

Data presented in Fig. 8A and B indicate that the land equivalent ratio (LER) values for all spatial arrangements of garlic-faba bean intercrop exceeded 1.0 in both seasons in both seasons, demonstrating a consistent and quantifiable land-use efficiency over sole cropping systems. Across all configurations, the relative yield of faba bean (RY_faba_) was greater than the relative yield of garlic (RY_garlic_). This asymmetry is primarily attributable to the experimental design, which maintained the faba bean population at 100% of its sole-crop density, while the garlic was intercropped at a reduced density of 33.3–50%. Consequently, faba bean faced less intraspecific competition and could more fully express its yield potential within the intercrop. The results further reveal a positive relationship between garlic proportion and system productivity. LER values increased progressively as the proportion of garlic was increased from one to three per planting ridge. This suggests a synergistic effect where a greater garlic component enhanced overall system productivity. Most remarkably, the 2RF:2RG pattern, which incorporated the highest garlic density, achieved the maximum LER values (1.58 and 1.65 in the first and second seasons, respectively). This pattern likely optimized resource partitioning by balancing the canopy architecture and minimizing interspecific competition for light and soil resources. Nonetheless, the 2SF:1LG arrangement, with the lowest relative garlic density, recorded the lowest LER values (1.15 and 1.21), underscoring the critical role of component crop density in optimizing land equivalence. The negative competitive effect observed in 2SF:1LG pattern can be attributed to intensified interspecific competition for above-ground resources, especially light, as the single garlic row was heavily shaded by two adjacent faba bean rows. Overall, the garlic-faba bean intercrop proved to be an advantageous system, enhancing land-use efficiency with LER values consistently greater than 1.0^12^. These results were consistent with the findings of previous reports^11,25^.

The land equivalent coefficient (LEC) is recognized as a particularly informative intercropping index because it precisely quantifies the nature and strength of the interspecific interaction between the component crops. Unlike more generalized metrics, such as the LER, which measures the aggregate land-use advantage without isolating the specific effects of crop-to-crop facilitation or competition, the LEC delineates the individual contributions of each species to overall system productivity^31^. In this study, the LEC values for all spatial arrangements exceeded the threshold of 0.25 in both seasons (Fig. 8A and B). Values above this benchmark demonstrate a genuine yield advantage of intercropping over sole cropping, indicating that the combined yield of both species in the mixture utilized land more efficiently than their separate monocultures. Notably, the productivity of these interactions varied significantly with spatial design The alternating 2RF:2RG pattern demonstrated superior system performance, achieving the highest LEC values (0.61 and 0.67 in 2023/24 and 2024/25, respectively). This suggests a more complementary and synergistic interaction between the two crops within this configuration. In contrast, the 2SF:1LG arrangement yielded the lowest LEC values (0.28 and 0.32), indicating a less efficient interaction likely dominated by competitive effects. This disparity underscores how spatial geometry directly influences the balance of interspecific competition and facilitation, thereby determining the overall productivity efficiency of the intercrop system. Abo El-Kassem and El-Shaieny^25^ also observed higher productivity and LEC values in faba bean-onion intercropping systems.

The competitive ratio (CR) analysis exhibited a distinct competitive hierarchy within the garlic-faba bean intercrop system (Fig. 8C and D). Across both seasons, the mean competitive ratio for faba bean (CR_a_) was 0.72, while that for garlic (CR_b_) was 1.40. A CR value > 1 signifies a competitively dominant species, confirming that garlic was the dominant competitor in this association, outperforming faba bean in capturing shared resources. Three primary reasons may explain this outcome. First, in legume-non-legume intercrops, applied nitrogen preferentially stimulates the growth of the non-legume (garlic), which has a higher nitrogen demand. Faba bean, capable of biological nitrogen fixation, is less aggressive in competing for soil N, thereby lowering its competitive ratio^12^. Second, a 30-day planting advantage provided garlic with a substantial head start, facilitating the establishment of a more extensive root system and the pre-emptive capture of soil nutrients and water before faba bean emergence. This temporal niche differentiation preferentially boosted resource acquisition and yield for garlic^12^. Third, the experimental design used a lower proportional density of garlic (33.3–50% of its sole crop stand) relative to a full-density faba bean stand. This asymmetric planting results in a higher CR for the under-represented species, as each individual plant has access to a relatively larger resource zone, strengthening its per-plant competitive ability^53^. A similar conclusion was also reached by Thapa et al.^54^, who found who reported a similarly low CR for pea (0.75) intercropped with a competitively dominant garlic (CR = 1.33). Finally, the results illustrated a consistent competitive hierarchy with garlic as the dominant species. Nevertheless, the intensity of competition was not statistically significantly modulated by the specific spatial arrangements tested, as reflected in the non-significant differences in CR values across all treatments and seasons.

**Table 7:** Analysis of variance for the effect of spatial arrangements on land equivalent ratio and coefficient and competitive ratio in garlic and faba bean intercropping system. *, **, *** = Significant at *p* ≤ 0.05, *p* ≤ 0.01, and *p* ≤ 0.001, respectively, ns = non-significant.

Source of variance	d.f	RY_Faba_	RY_Garlic_	LER	LEC	CR_a_	CR_b_	CR
1st season (2023/2024)								
Blocks	2	0.005**	0.001^ns^	0.009*	0.003^ns^	0.002^ns^	0.013^ns^	0.004^ns^
Arrangement	3	0.01***	0.07***	0.139***	0.082***	0.008^ns^	0.032^ns^	0.008^ns^
Error	6	1.44	0.001	6.94	7.27	0.003	0.016	0.005
F-test		76.23	65.25	201.14	112.96	2.709	1.933	1.67
*p* value		0.0007	0.0009	0.0001	0.0003	0.180	0.258	0.296
CV (%)		1.37	7.13	1.94	6.30	7.84	9.22	3.30
2nd season (2024/2025)								
Blocks	2	1.333^ns^	0.002^ns^	0.003 ^ns^	0.002^ns^	0.005 ^ns^	0.021^ns^	0.005^ns^
Arrangement	3	0.011**	0.09***	0.16***	0.11***	0.006 ^ns^	0.02^ns^	0.005^ns^
Error	6	3.17	8.66	7.44	4.61	0.003	0.01	0.003
F-test		36.11	111.84	213.55	227.73	1.63	1.74	1.62
*p* value		0.003	0.0003	0.0001	0.0001	0.304	0.28	0.31
CV (%)		1.97	5.88	1.94	4.69	8.21	7.78	2.55

#### Aggressivity, relative crowding coefficient, and actual yield loss

According to the ANOVA results (Table 8), spatial arrangement had a highly significant effect (*p* < 0.001) on all measured competitive indices, including Ag_Faba_, Ag_Garlic_, K_ab_, K_ba_, RCC, AYL_F_, and AYL_G_ in the first and second seasons. The sole exception was the effect on AYL, which was significant at *p* < 0.01 in both seasons.

**Fig. 9:**
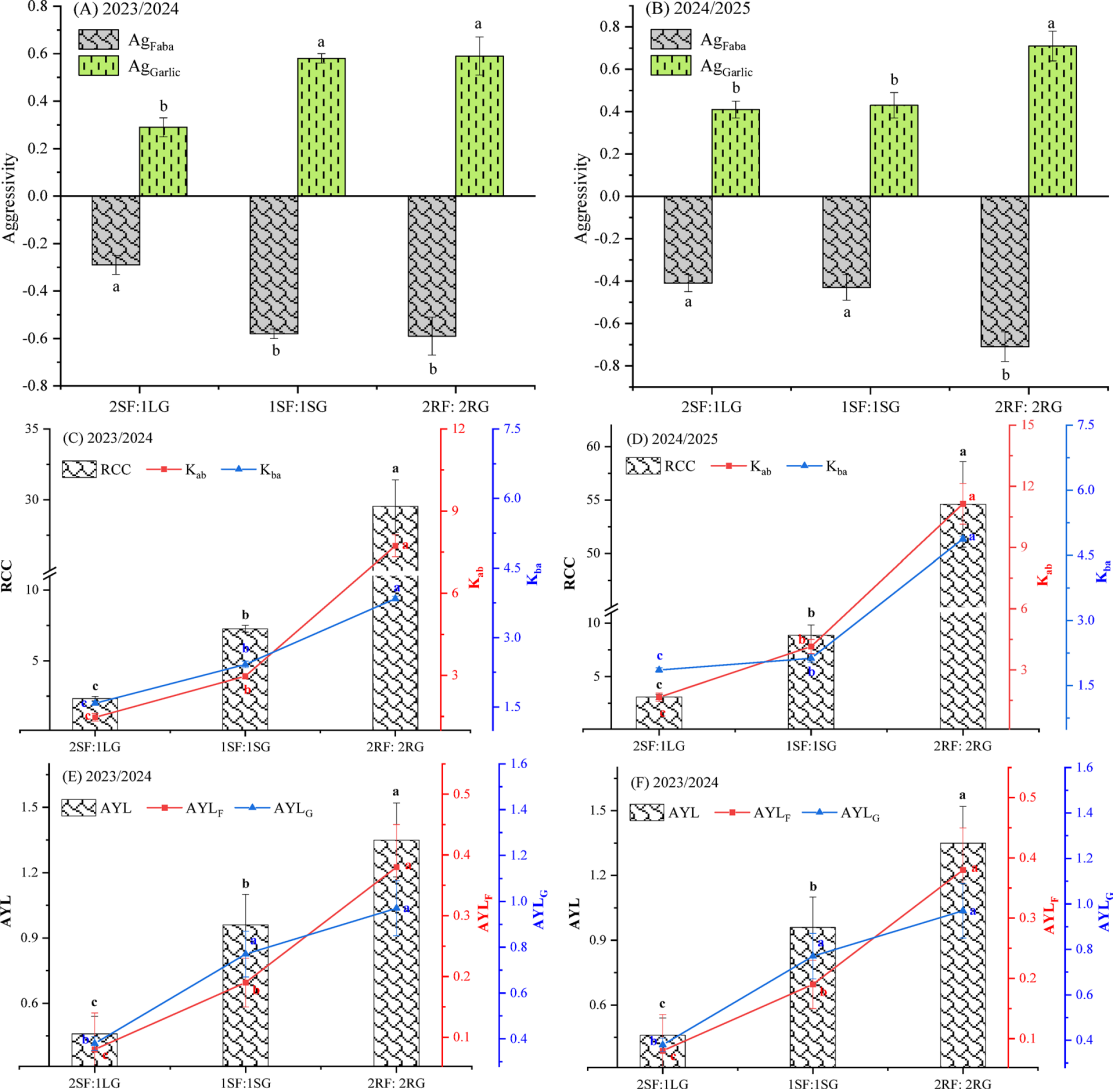
Effect of spatial arrangements on the aggressivity (**A**, **B**), relative crowding coefficient (**C**, **D**), and actual yield loss (**E**, **F**) in garlic and faba bean intercropping system. Error bars represent the standard deviation of three determinations. Different letters on bars denote significant differences based on Tukey’s test at *p* < 0.05.

Aggressivity measures the relative competitive ability among component crops in an intercropping system. Higher numerical values denote a greater disparity in competitive ability and a larger difference between the actual and expected yield of both crops. A positive value indicates the dominant species, while a negative value signifies the dominated species. In this study, the aggressiveness value was positive for intercropped garlic across all spatial arrangements, whereas it was negative for faba bean in the two growing seasons (Fig. 9A and B). These results demonstrate that garlic was the dominant crop, while and faba bean was the dominated one. The interpretation of this result can be attributable to the reduced plant density of garlic in the intercropping system, which minimized the negative effects of intraspecific competition, and subsequently enhanced its aggressivity. Additionally, the growing two faba bean plants per hill intensified root competition, which dramatically impaired the competitive ability of faba bean against garlic. Rashwan and Zeneldin^55^ found that soybean was the dominant species over maize. They also observed that maize exhibited negative aggressivity when two plants were grown per hill compared to one plant per hill in sole planting.

The relative crowding coefficient (RCC) varied significantly among intercropping arrangements in both seasons (*p* < 0.05) (Fig. 9C and D). In all cases, RCC values exceeded 1.00, indicating overall yield advantage under intercropping. The lowest RCC values (2.33 and 3.05 in the first and second season, respectively) were recorded in the 2SF:1LG arrangement, where a single line of garlic was grown between two strips of faba bean. This can likely be attributed to reduced intraspecific competition in garlic, as reflected by its lower garlic-specific competition value (1.58 and 1.86, in the first and second respectively) relative to other spatial configurations. In contrast, the 2RF:2RG alternating ridge pattern produced the highest RCC values (29.54 and 54.60 in both seasons). These results suggest that the canopy architecture of the alternating ridge system created a more favorable intercropping environment. The physical separation between crop rows on adjacent ridges may have improved light distribution and penetration for both species, thereby reducing interspecific competition for light and enhancing overall system efficiency and productivity. Likewise, Abo El-Kassem and El-Shaieny^25^ recorded RCC values above 1.00 for a faba bean-onion intercrop, indicating a yield advantage over sole cropping.

As shown in Fig. 9E and F, the actual yield loss (AYL) index provided a more precise quantification of competitive interactions between intercrops compared to other indices. Specifically, the AYL values for faba bean (AYL_F_) were positive yet consistently lower than those for garlic (AYL_G_) across all spatial arrangements in both growing seasons. This finding is in agreement with the trends observed in the competitive ratio (CR) and aggressivity indices, collectively indicating that faba bean was less resilient to yield reduction under intercropping conditions than garlic. This outcome was probably due to the fact that the earlier-sown crop (garlic) had an initial advantage in establishing and competing for the available resources. Crucially, the total AYL values were significantly positive (*p* < 0.05) across all spatial arrangements in both seasons, signifying a net overall yield benefit for the intercropping system compared to sole cropping and demonstrating the principle of complementarity. The highest total AYL values (1.35 and 1.45) were recorded for the 2RF:2RG alternating ridge arrangement during the 2023/24 and 2024/25 growing seasons, respectively. In contrast, the lowest values (0.45 and 0.56) were observed in the 2SF:1LG pattern. This further suggests that the alternating ridge layout was particularly effective in optimizing resource partitioning and minimizing negative competitive interactions, thereby maximizing the overall productivity benefit of the intercropping system. Similar findings have been reported previously^25^.

**Table 8:** Analysis of variance for the effect of spatial arrangements on aggressivity, relative crowding coefficient, and actual yield loss in garlic and faba bean intercropping system. *, **, *** = Significant at *p* ≤ 0.05, *p* ≤ 0.01, and *p* ≤ 0.001, respectively, ns = non-significant.

Source of variance	d.f	Ag_Faba_	Ag_Garlic_	K_ab_	K_ba_	RCC	AYL_F_	AYL_G_	AYL
1st season (2023/2024)									
Blocks	2	0.006*	0.006*	0.132^ns^	0.008**	1.687^ns^	0.010 **	0.028*	0.048*
Arrangement	3	0.087***	0.087***	32.047***	3.914***	630.37***	0.069 ***	0.270***	0.597**
Error	6	9.33	9.33	0.027	2.33	0.92	0.0004	0.003	0.002
F-test		93.32	93.32	1156.95	167777.7	680.67	172.75	72.35	293.65
*p* value		0.0004	0.0004	0.0001	0.0001	0.0001	0.0001	0.0007	0.0001
CV (%)		6.27	6.27	4.10	0.58	7.37	9.23	8.64	4.88
2nd season (2024/2025)									
Blocks	2	0.009**	0.009**	2.43***	0.01**	9.01^ns^	7.77^ns^	0.003*	0.05^ns^
Arrangement	3	0.08***	0.08***	72.46***	8.37***	2389.53***	0.06***	0.295***	0.62**
Error	6	2.33	2.33	0.01	4.33	3.97	2.77	2.33	0.02
F-test		361.71	361.71	7246.53	19333.6	601.01	2299.6	1266.85	30.01
*p* value		0.0001	0.0001	0.0001	0.0001	0.0001	0.0001	0.0001	0.0004
CV (%)		2.95	2.95	1.76	0.70	8.98	3.00	1.99	15.34

### Economic evaluation

Economically, the total return and total production costs for the garlic-faba bean intercropping system were significantly higher than sole cropping of faba bean alone in both seasons (*p* < 0.05) (Table 9). Despite these increased costs, the net return, reflecting actual profitability, was substantially higher for all intercropping treatments compared to either sole crop of faba bean or garlic. The economic advantage of intercropping was further confirmed by benefit-cost ratio (BCR), which exceeded 1.00 across all faba bean intercropping arrangements. Market prices during the study period were $741 and $764 per ton for faba bean seed, and $317 and $301 per ton for garlic bulb yield in the first (2023/24) and second (2024/25) seasons, respectively. Among the spatial arrangements tested, the alternating ridge pattern (2RF:2RG) proved the most economically efficient, recording the highest values for gross return ($6,093.3 and $6,706.7 ha^−1^), net return ($3,260.3 and $3,980.7 ha^−1^), and BCR (2.15 and 2.46) in the respective seasons. These superior economic outcomes can be directly attributed to the higher overall land productivity (LER > 1, Fig. 8A and B) and yield stability achieved through complementary resource use in this arrangement, which translated into greater marketable yield per unit area. The 2RF:2RG layout likely optimized resource capture and reduced interspecific competition, thereby enhancing the combined marketable yield and maximizing revenue per unit area. El-Shamy and Abd El-Aty^47^ and also Abo El-Kassem and El-Shaieny^25^ reached a similar conclusion. The consistent profitability, evidenced by BCR values above unity and enhanced net returns, underscores that intercropping garlic with faba bean is not only agronomically efficient but also financially viable, improving farmers income compared to conventional sole cropping systems.

**Table 9:** Economic evaluation of garlic-faba bean intercropping under different spatial arrangements.

Treatment	Return of faba bean ($.ha^−1^)	Return of garlic ($.ha^−1^)	Total return ($.ha^−1^)	Total cost ($.ha^−1^)	Net return ($.ha^−1^)	B:C ratio
1st season (2023/2024)						
SF	3155.3^a^	–	3155.3^d^	2015.0^d^	1140.3^d^	1.57^c^
SG	–	4844.3^a^	4844.3^b^	3720.0^a^	1124.3^d^	1.30^d^
2SF:1LG	2551.0^c^	1671.0^d^	4221.6^c^	2560.0^c^	1661.6^c^	1.65^c^
1SF:1SG	2816.0^b^	2143.0^c^	4959.0^b^	2560.0^c^	2399.0^b^	1.94^b^
2RF:2RG	2914.3^b^	3179.0^b^	6093.3^a^	2833.0^b^	3260.3^a^	2.15^a^
2nd season (2024/2025)						
SF	3276.3^a^	–	3276.3^d^	1938.0^d^	1338.3^d^	1.69^d^
SG	–	5084.33^a^	5084.3^b^	3580.0^a^	1504.3^d^	1.42^e^
2SF:1LG	2727.0^c^	1929.00^c^	4655.7^c^	2467.0^c^	2188.7^c^	1.89^c^
1SF:1SG	3025.3^b^	2109.67^c^	5135.3^b^	2466.0^c^	2669.3^b^	2.08^b^
2RF:2RG	3108.0^ab^	3598.67^b^	6706.7^a^	2726.0^b^	3980.7^a^	2.46^a^

### Correlation analysis

**Fig. 10:**
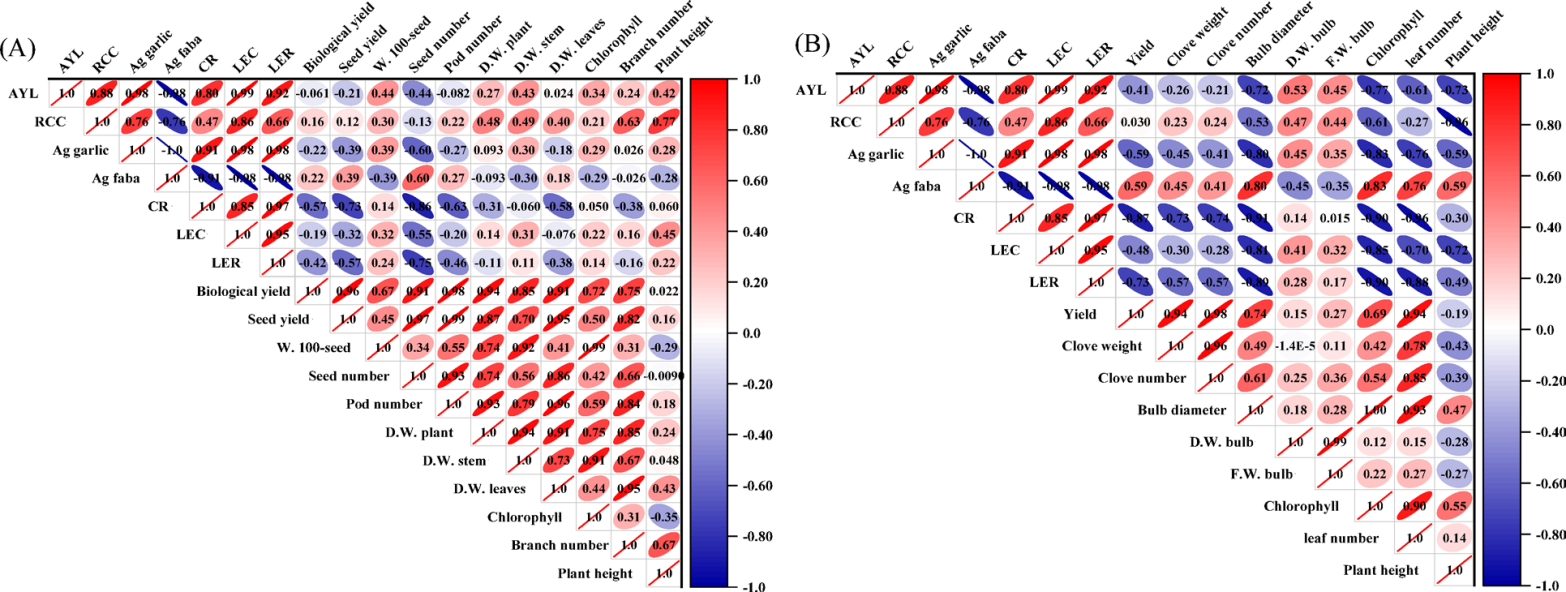
Pearson’s correlation analysis of growth, yield, and competition parameters for faba bean (**A**) and garlic (**B**).

To visualize the interrelationships among growth, yield, and competition parameters, Pearson’s correlation analysis was performed for faba bean and garlic across the intercropping systems (Fig. 10). In faba bean (Fig. 10A), plant height showed considerable positive correlations with the number of branches (*r* = 0.67) and the relative crowding coefficient (RCC) (*r* = 0.77). This suggests that taller plants were more vigorous individuals within the intercropping systems. In the mixed canopy, height is a crucial trait for light foraging, allowing faba bean to overtop its companion crop. The success in this vertical competition may have supplied the resources necessary for lateral expansion, thereby increasing branch number and further intensifying light interception^56^. This synergistic enhancement in canopy architecture led to greater resource uptake and biomass accumulation, which is quantitatively reflected in a higher RCC. Moreover, chlorophyll content was strongly and positively interrelated with dry weight of stem (*r* = 0.91), dry weight of plant (*r* = 0.75), and biological yield (*r* = 0.72). These findings are primarily attributable to the intensification of photosynthesis resulting from higher chlorophyll content, which produced more carbon assimilates for structural growth, particularly in stems and leaves. The resulting increase in photosynthetic tissue likely created a larger, more efficient photosynthetic apparatus (i.e., chlorophyll-containing tissue), thereby boosting the overall photosynthetic capacity of the plants and consequently leading directly to greater dry matter production and, ultimately, a higher biological yield. Similar associations among chlorophyll content and dry matter production have been reported^57^. Increases in dry weight of plant strongly explained the variation in key yield components, accounting for 74–94% of the enhancement in seed number per pod, 100-seed weight, pod number per plant, seed yield and biological yield. This demonstrates that vegetative growth, as represented by dry weight of plant, serves as a critical source of assimilates, with its increase directly strengthening the source capacity to supply developing sinks (seeds and pods), thereby maximizing both the yield components and the final harvestable output in faba bean^58^.

Regarding garlic, chlorophyll content exhibited significant negative correlations with system AYL (*r* = − 0.77), CR (*r* = − 0.90), RCC (*r* = − 0.61), and LER (*r* = − 0.91) (Fig. 10B). These relationships suggest that, where faba bean was highly dominant, garlic likely faced intense shading. This low-light stress may have triggered a compensatory shade-acclimation response, improving chlorophyll content to maximize light capture. Maintaining this high chlorophyll content under stress represented a significant physiological adaptation, potentially diverting nitrogen and energy away from bulb development and below-ground competition. Consequently, the highest garlic chlorophyll levels were associated with its poorest competitive performance and the lowest contribution to overall system productivity, which mechanistically explains the observed negative correlations. Furthermore, the increase in chlorophyll content was positively correlated with leaf number (*r* = 0.90), highlighting its beneficial role in garlic vegetative growth. This relationship was primarily driven by an enhanced photosynthetic capacity under high chlorophyll levels, which boosted the carbon assimilates and energy available for direct allocation to the production and expansion of new leaves. These finding was consistent with the observation of. Most importantly, positive associations were observed between leaf number and clove weight, clove number, and bulb diameter (*r* = 0.78–0.93), demonstrating its substantial contribution to the increase in final yield. This finding was mainly explained by the source-sink relationship, whereby a greater leaf number enhanced photoassimilates production^59^. These assimilates were then directly allocated to support the growth and development of the bulb and cloves, thereby improving yield and its components. In conclusion, the statistical analysis indicated that the growth traits of faba bean and garlic were strongly linked to their respective yield and competition parameters across the intercropping patterns.

### Principal component analysis (PCA)

**Fig. 11:**
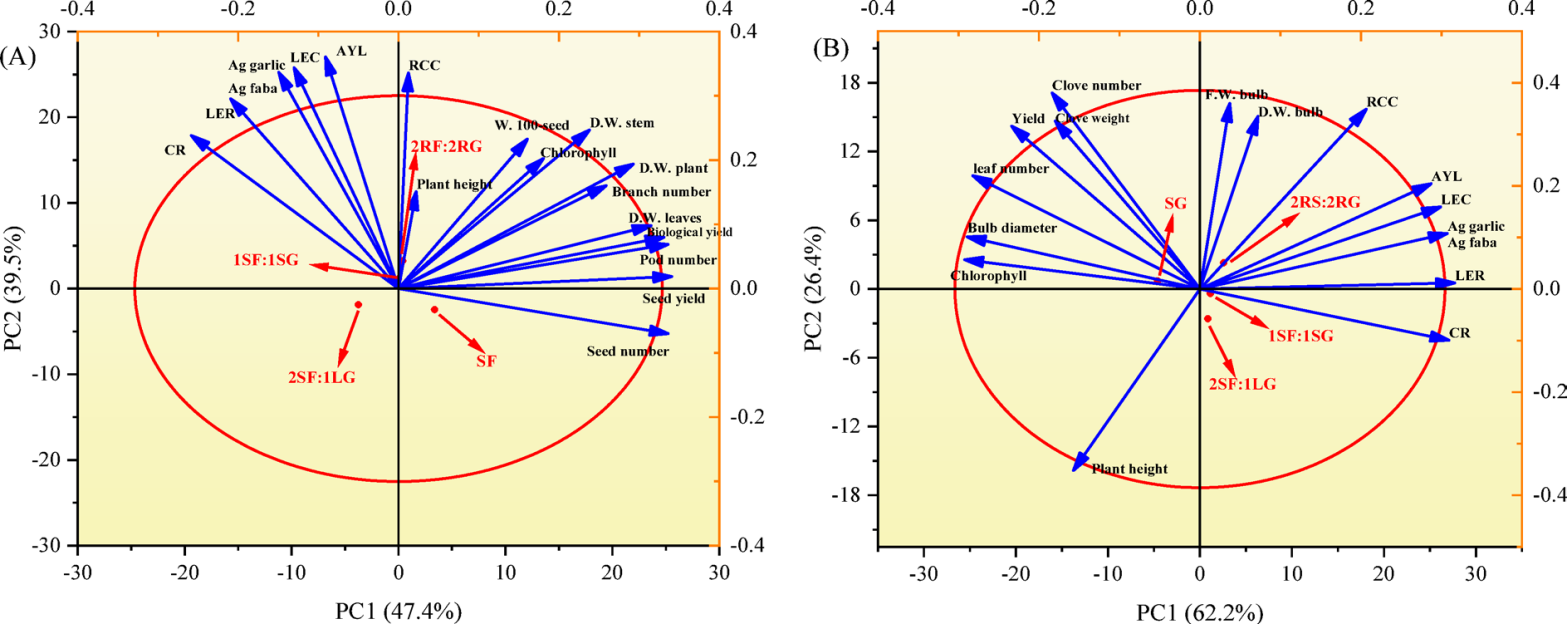
Principal component analysis of growth, yield, and competition parameters for faba bean (**A**) and garlic (**B**) under different intercropping systems.

PCA was employed to synthesize and interpret the effects of various intercropping arrangements on growth, yield, and competition parameters for both faba bean and garlic. For faba bean (Fig. 11A), the first two principal component (PC1 and PC2) explained 47.40% and 39.50% of the total variance, respectively. Their cumulative explanatory power of 86.90% captured a substantial portion of the underlying structure of the data, providing a reliable basis for interpreting similarities and distinctions among the intercropping treatments^60,61^. Based on the computed loading values and factor scores, the intercropping systems and their corresponding parameters were distinctly categorized into four groups. The 2RF:2RG planting arrangement was positioned on the positive side of both PC1 and PC2. This placement indicates a strong positive association with plant height, chlorophyll content, branch number, pod number, 100-seed weight, dry weight of stem, leaves, and total plant, seed yield, biological yield, and relative crowding coefficient. In essence, the variations in growth, yield, and competitive ability (as quantified by the relative crowding coefficient) of faba bean were strongly correlated with this specific intercropping pattern. This correlation suggests that the 2:2 strip configuration likely reduced direct interspecific competition for water and nutrients between faba bean and garlic more effectively than other planting patterns. This reduction can be ascribed to the complementary root architectures and nutrient demands of the two species. Such complementarity may have enabled faba bean to exploit a distinct soil niche, thereby increasing overall resource-use efficiency. With reduced competition from garlic, faba bean plants could consequently allocate more resources to vegetative growth and seed production, accounting for the observed enhancements in plant height, dry matter accumulation, and yield components. The second group, representing the 1SF:1SG intercropping arrangement and located in the upper left-hand quadrant, was characterized by metrics for interspecific competition, including the aggressivity of both species, actual yield loss, competition ratio, and the land equivalent ratio and coefficient. This clustering implies that this planting arrangement fostered a strongly competitive environment without a decisive advantage for either species, resulting in suboptimal performance for both. Conversely, the 2SF:1LG arrangement, located on the negative sides of both principal components, showed no strong association with any of the measured parameters. The fourth group, corresponding to the sole faba bean (SF) treatment and positioned in the lower right-hand quadrant, was primarily defined by a high seed number. This result indicates that the absence of interspecific competition in the monoculture allowed faba bean plants to allocate a greater proportion of resources to reproductive development, thereby maximizing seed number.

For garlic (Fig. 11B), PC1 and PC2 contributed to 62.20% and 26.40% of the total variance, respectively, yielding a cumulative explained variance of 88.60%. The first cluster, containing the 2RS:2RG arrangement, fell on the positive sides of both components and was associated with interspecific competition parameters (i.e., the aggressivity of both species, actual yield loss, relative crowding coefficient, and the land equivalent ratio and coefficient) as well as with fresh and dry weight of bulb. These interrelationships reveal that the 2RS:2RG configuration strongly influenced competitive dynamics and enhanced dry matter accumulation in garlic bulbs. Furthermore, the sole garlic (SG) treatment was located in the upper left-hand quadrant, exhibiting distinct associations with chlorophyll content, leaf number, bulb diameter, clove number and weight, and total yield. This outcome indicates that, in the absence of interspecific competition, garlic plants allocated a greater proportion of captured resources (e.g., water, nutrients, and photosynthates) to their own growth and development rather than to competitive traits. This optimized resource allocation enhanced photosynthetic capacity (maximizing light capture and carbon assimilation) and facilitated the efficient translocation of assimilates to the bulb, thereby increasing bulb development and final yield. Surprisingly, plant height was isolated in a third group on the negative sides of PC1 and PC2 and showed no clear association with any specific intercropping pattern. Such finding demonstrates that plant height responded independently of the competitive and yield-related traits that defined the other clusters. The 2SF:1LG and 1SF:1FG systems were positioned in the lower right-hand quadrant with the competition ratio. The observed relationship is attributable to their structural imbalance inherent in these arrangements, which amplified competition for a primary limiting resource (e.g., light or soil nitrogen). This intense competitive interaction became a defining feature of their multivariate profile in the PCA.

#### Radar plot analysis

**Fig. 12:**
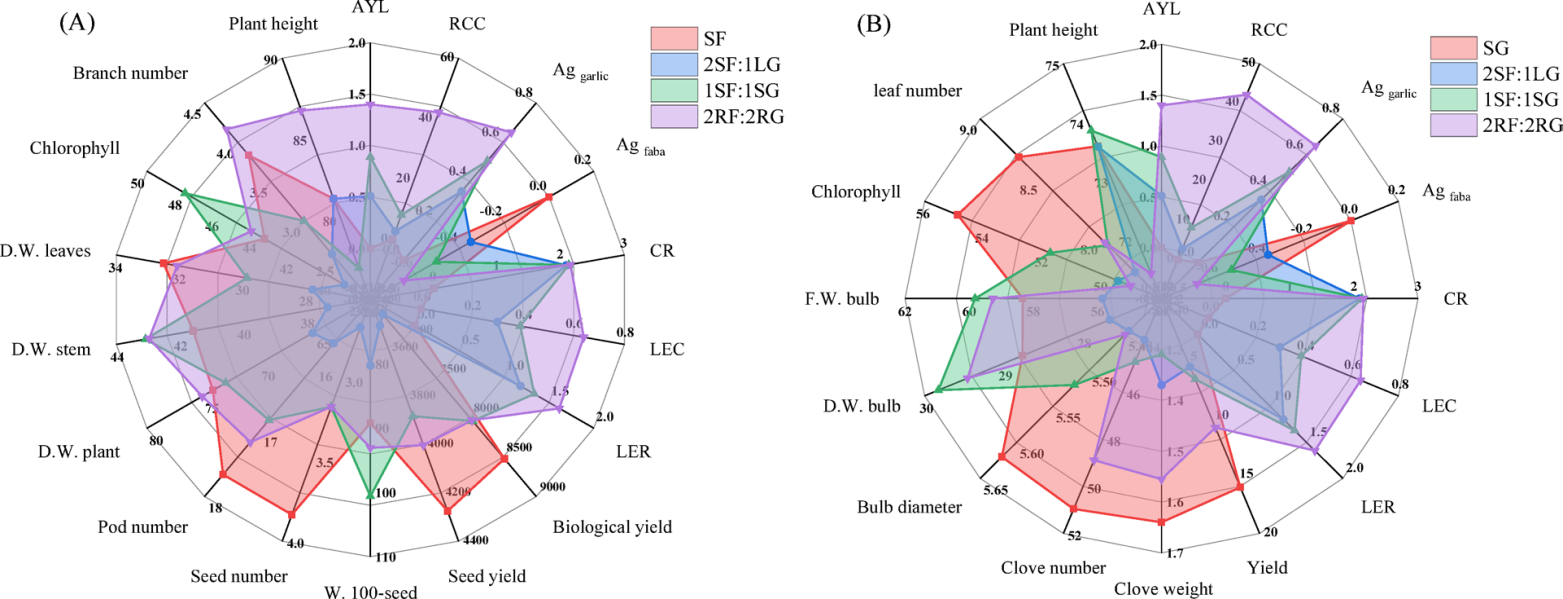
Radar plots comparing growth, yield, and competition parameters for (**A**) faba bean and (**B**) garlic across different intercropping systems.

Radar plots are a powerful tool for simultaneous visualization and comparative assessment, enabling the intuitive identification of dominant patterns among many parameters^62^. The analysis illustrated a clear hierarchy in the influence of the intercropping systems on growth, yield, and competition parameters for faba bean and garlic (Fig. 12). For this visualization, data for each trait were normalized to a 0–100% scale to reflect their relative magnitude under the different treatments. For faba bean (Fig. 12A), the sole faba bean (SF) treatment exhibited a predominant impact on most growth and yield traits, accounting for about 80–89% of the contribution. This dominance was observably evident for dry weight of leaves, pod number per plant, seed number per pod, seed yield, and biological yield. Moreover, the 2RF:2RG configuration contributed substantially (~ 76 to 87%) to several structural and competitive traits, most notably branch number, plant height, the land equivalent ratio (LER), the land equivalent coefficient (LEC), and the aggressivity of garlic (Ag _garlic_). This suggests that the alternating two-ridge arrangement created distinct territorial zones, reducing intraspecific competition and fostering complementary light capture. These effects enhanced resource-use efficiency and microclimate, which led to greater system productivity (higher LER and LEC). Simultaneously, the arrangement established a stable competitive hierarchy, with garlic exhibiting clear aggressivity. This demonstrates that the specific planting configuration successfully transformed a typically antagonistic intercrop dynamic into a more productive and stable agroecological system. The 1SF:1SG pattern also had a major influence on chlorophyll content and 100-seed weight, explaining ~ 78 and 84% of their variation, respectively. In contrast, the 2SF:1LG system showed minimal contributions to all growth, yield, and competition parameters. The ineffectiveness of the 2SF:1LG system can be attributed to a spatial imbalance that induced intense competition without enabling significant complementarity. The single central line of garlic provided insufficient competitive or facilitative mass to alter resource dynamics, while the two outer faba bean rows likely overshadowed and outcompeted it for key resources (e.g., light, water, and nutrients), severely suppressing garlic performance. This resulted in a state of diffuse, unproductive competition, ultimately explaining the negligible statistical contribution of this system to all measured traits.

Figure 12B shows that the sole garlic (SG) pattern consistently exhibited the highest contribution (~ 79 to 90%) across most growth and yield parameters of garlic, including leaf number, chlorophyll content, bulb diameter, clove number, clove weight, and total yield. Nonetheless, its contribution to the fresh and dry weight of bulbs was lower than that of the 1SF:1SG and 2RF:2RG systems. This dominance was probably linked to the resource exclusivity of a monoculture, where garlic plants were grown without competitive pressure from a companion species. Such exclusive access to essential resources may have optimized key physiological processes, such as photosynthesis, nutrient uptake, and assimilate partitioning, directly translating into enhanced vegetative growth and reproductive output. Furthermore, a strong pattern was observed for the LER, LEC, Ag _garlic_, CR, relative RCC, and AYL, in which the 2RF:2RG arrangement was the primary contributor, explaining ~ 78 to 87% of the variation across these competitive traits. The analysis also revealed that the 1SF:1SG configuration was the primary explanatory factor for plant height, fresh bulb weight, and dry bulb weight, with contributions ranging from 72 to 94%. The influence of the 2SF:1LG system was highly variable, ranging from 18% to 78% across the majority of growth, yield, and competition indices. Overall, however, it demonstrated the lowest consistent contribution among all intercropping systems.

### Heatmap analysis

**Fig. 13:**
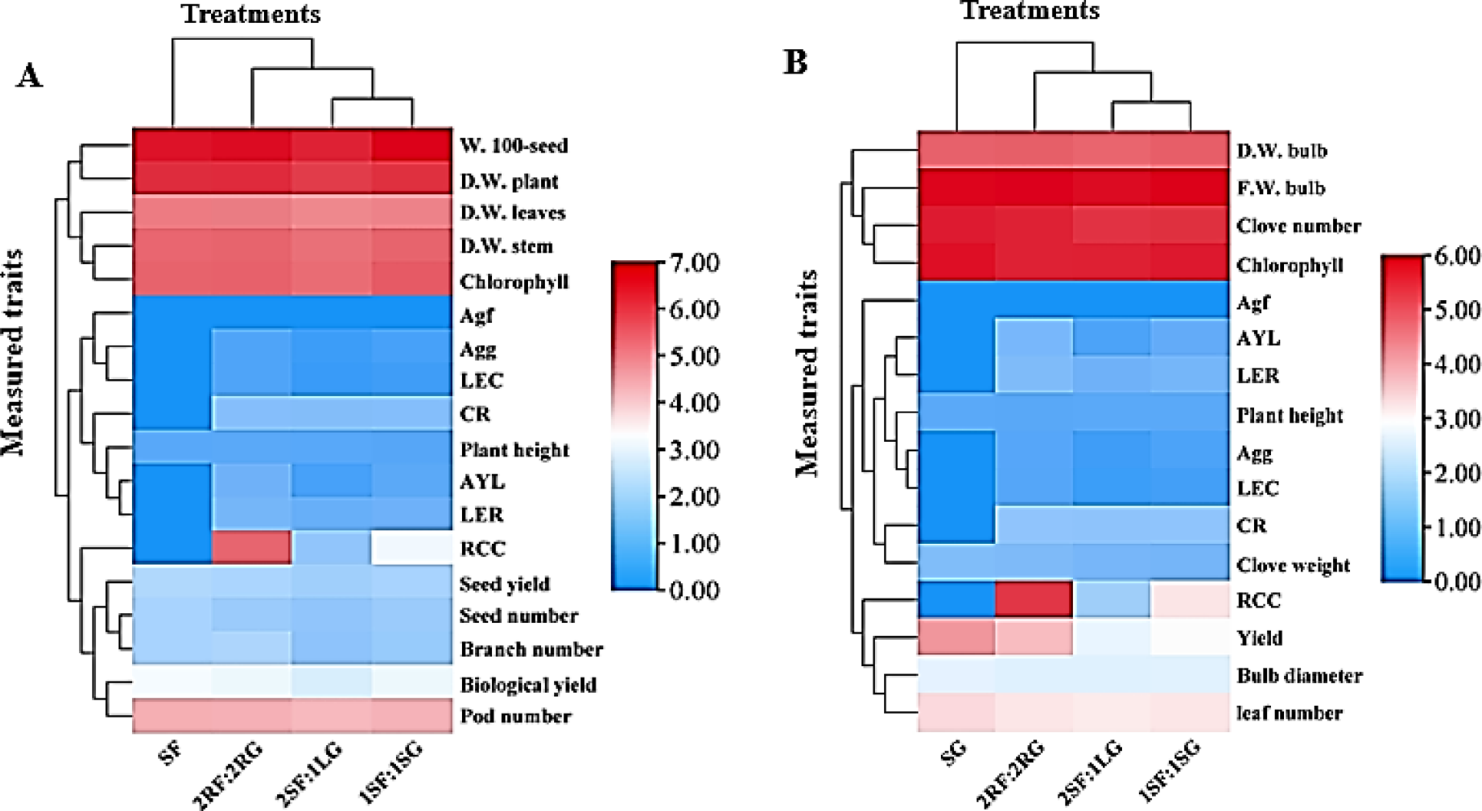
Hierarchical clustering heat map showing the effects of different intercropping systems on growth, yield, and competition parameters for (**A**) faba bean and (**B**) garlic.

The interrelationships between different intercropping systems and the comprehensive dataset of faba bean and garlic traits (growth, yield, and competition parameters) were elucidated using a clustering heatmap analysis (Fig. 13). The heatmap revealed clear groupings, indicating distinct patterns in how the measured traits responded. For faba bean (Fig. 13A), traits including chlorophyll content and the dry weights of leaves, stems, and the whole plant, along with 100-seed weight, formed a distinct cluster characterized by low values under the 2SF:1LG pattern. The coordinated decline in photosynthetic and vegetative traits indicates a systemic stress response, likely induced by the suboptimal microenvironment of the 2SF:1LG pattern. Within this arrangement, combined abiotic stresses (such as altered light availability and root-zone interactions with garlic) downregulated primary metabolism. This led to reduced carbon fixation and assimilation, which directly limited vegetative biomass and, ultimately, the resources allocated to seed filling, resulting in lower seed weight. Notably, despite being separated into different clusters, all competitive parameters (RCC, LEC, LER, CR, and AYL) exhibited their maximum values under the 2RF:2RG arrangement, highlighting a consistent agronomic advantage of this pattern. Moreover, the sole cropping system of faba bean produced the clearest cluster of high-value responses, characterized by the most pronounced positive effects on branch number, pod number, seed number, seed yield, and biological yield. This performance was primarily linked to the absence of interspecific competition. In the sole crop, faba bean plants had unrestricted access to essential resources without facing competitive pressure from a companion species (i.e., garlic). This facilitated optimal resource partitioning towards branch initiation and reproductive development, enhancing pod set and seed number, which ultimately maximized both seed and biological yield.

Concerning garlic (Fig. 13B), the fresh bulb weight, dry bulb weight, clove number, and chlorophyll content formed a primary response cluster. Within this cluster, a gradient was visible: dry bulb weight peaked under the 1SF:1SG pattern, whereas fresh bulb weight, clove number, and chlorophyll content formed a sub-cluster, reaching their maximum values under the sole garlic (SG) cropping system. Furthermore, a coherent cluster comprising all measured competitive parameters (RCC, LEC, LER, CR, and AYL) underscores the superiority of the 2RF:2RG pattern, as this treatment uniquely elicited peak values for every metric. This visualization confirms that among the tested patterns, 2RF:2RG is the most agronomically effective, providing a clear data-driven recommendation for achieving the highest land-equivalent and competitive performance. The heatmap analysis also confirms that the SG treatment yielded the best overall results, a finding driven by strong, congruent improvements in leaf number, bulb diameter, and total yield, together forming a distinct cluster. This suggests that the SG system created an optimal, low-stress environment where photosynthate was efficiently partitioned towards bulb enlargement and yield. Overall, the heatmap analysis revealed that the 2RF:2RG treatment represented the most balanced and synergistic intercropping system, achieving optimal land-use efficiency and providing a competitive advantage for both faba bean and garlic simultaneously.

## Conclusions

This study investigated the effects of different spatial arrangements on the growth, yield-related traits, competitive indices, and profitability of faba bean-garlic intercropping. Results revealed that the synergistic interaction between these two crops enhanced the organic acid content in the faba bean rhizosphere. Remarkably, the 2SF:1LG pattern suppressed nearly all growth traits in both species, except for faba bean plant height, indicating that intense interspecific competition limited resources for biomass production. Among intercropping systems, the 2RF:2RG arrangement produced the highest faba bean and garlic yields. Moreover, the 2RF:2RG arrangement optimized biological efficiency, achieving the highest land use efficacy (indicated by LER and LEC) and relative crowding coefficient, and economic performance, with superior net returns and benefit-cost ratios. Notably, the interspecific competition analysis further confirmed garlic as the dominant competitor, exhibiting a higher competition ratio and aggressivity than the subordinate faba bean. In conclusion, the 2RF:2RG spatial arrangement is recommended for garlic-faba bean intercropping to achieve the highest yields and maximize land productivity and profitability.

## Data Availability

All data are included in this article, and any further information will be made available from the corresponding author on reasonable request.
